# New Early Cretaceous palaeomagnetic and geochronological results from the far western Lhasa terrane: Contributions to the Lhasa-Qiangtang collision

**DOI:** 10.1038/s41598-017-16482-3

**Published:** 2017-11-24

**Authors:** Weiwei Bian, Tianshui Yang, Yiming Ma, Jingjie Jin, Feng Gao, Shihong Zhang, Huaichun Wu, Haiyan Li

**Affiliations:** 1 State Key Laboratory of Biogeology and Environmental Geology, China University of Geosciences, Beijing, 100083 China; 2 School of Earth Sciences and Resources, China University of Geosciences, Beijing, 100083 China; 3 Guangzhou Institute of Geochemistry, Chinese Academy of Sciences, Guangzhou, 510640 Guangdong, China

## Abstract

To better constrain the Lhasa**-**Qiangtang collision, a combined palaeomagnetic and geochronological study of the far western Lhasa terrane was conducted on the Duoai Formation lava flows (~113–116 Ma), as well as on the Early Cretaceous Jiega Formation limestone. Following detailed rock magnetic, petrographical, and palaeomagnetic experiments, characteristic remanent magnetisation directions were successfully isolated from most samples using principal component analysis. The tilt-corrected direction groups yielded a palaeopole at 69.1°N, 319.8°E with A_95_ = 4.8° (N = 19). A primary origin for the magnetisation is consistent with positive fold tests. Our results from the Early Cretaceous units, combined with published palaeomagnetic data obtained from Cretaceous strata from the Lhasa and western Qiangtang terranes, show that these two terranes had already collided by the Early Cretaceous, the Lhasa terrane had a relatively east-west alignment, and it remained at a relatively stable palaeolatitude during the entire Cretaceous. Comparing the Cretaceous palaeolatitude calculated for the western Lhasa terrane with those from Eurasia and Mongolia suggests a latitudinal convergence of ~1400 ± 290 km and ~1800 ± 300 km, respectively, since the Early Cretaceous.

## Introduction

**Figure 1:**
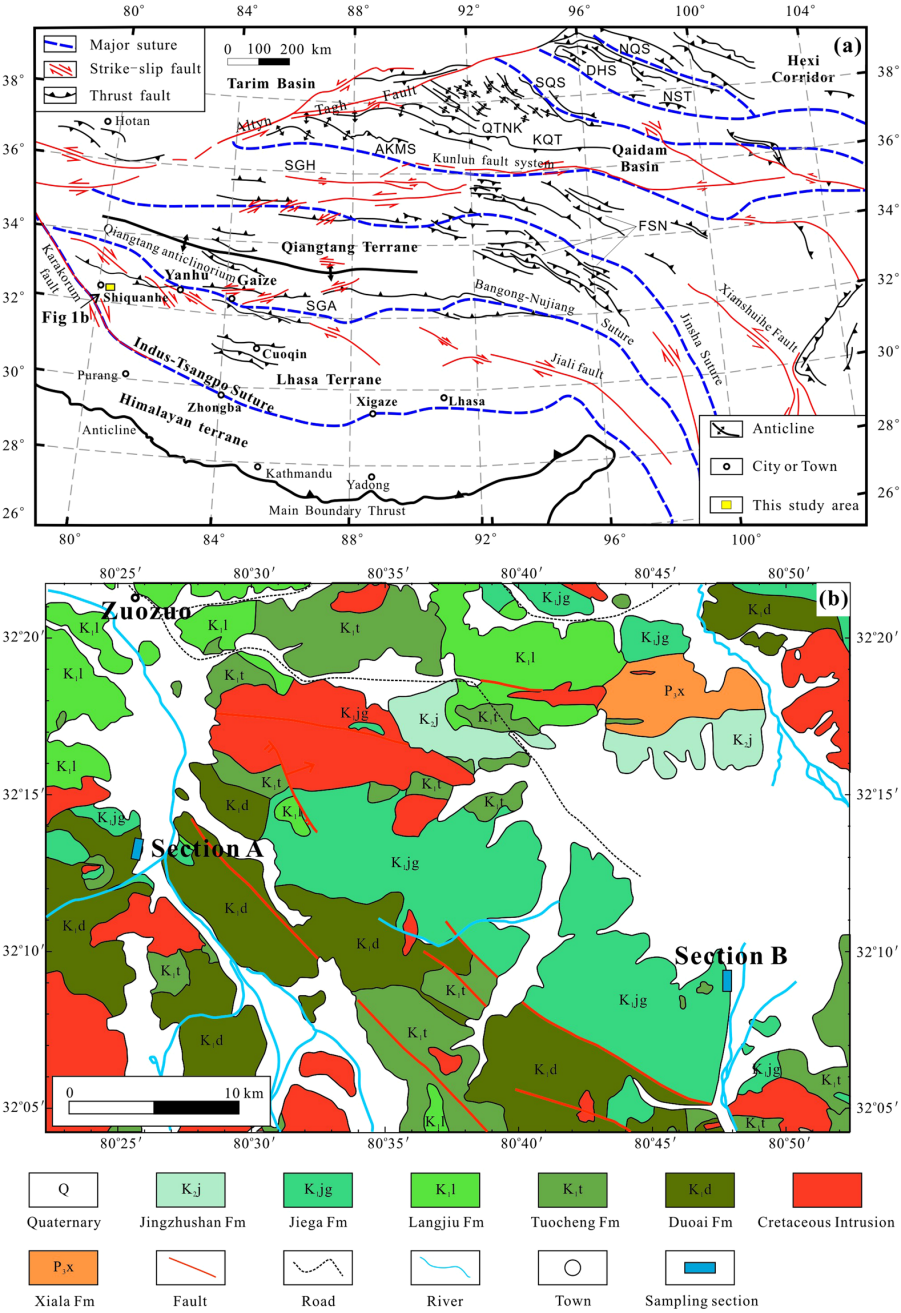
Geology and sampling location for this study. (**a**) Tectonic sketch map of central Asia modified after Yin and Harrison^1^. Abbreviations: AKMS, Ayimaqin-Kunlun-Muztagh suture; DHS, Danghe Nan Shan suture; FSN, Fenghuo Shan-Nangqian fold and thrust belt; KQT, Kunlun-Qaidam terrane; QTNK, Qimen Tagh-North Kunlun thrust system; NQS, North Qilian Suture; NST, Nan Shan thrust belt; SGA, Shiquanhe-Gaize-Ando thrust system; SGH, Songpan-Ganzi-Hoh Xil terrane; SQS, South Qilian suture; (**b**) Simplified geological map of the sampling area.

The Tibetan Plateau, known as the Earth’s third pole, is a complex amalgamation of several continental fragments that include, from south to north, the Himalaya, Lhasa, Qiangtang, Songpan-Ganzi, and Kunlun-Qaidam blocks (Fig. 1a). These continental fragments have gradually accreted to the stable Asian continent since the Early Palaeozoic^1^, and thus the record of their plate motions, collisions, and subsequent deformation plays a key role in understanding the formation and evolution of the Tibetan Plateau^2,3^. Those processes have a tremendous effect on global climate and the evolution of life^4^ and understanding the formation and evolution of the Tibetan Plateau would increase our knowledge of plate tectonic kinematics and climate change.

Palaeomagnetism is the only method that can quantify palaeolatitude and is thus instrumental in constraining the kinematic processes of terrane movement^5–12^. The Lhasa terrane is located between the Bangong-Nujiang suture zone to the north and the Indus-Yarlung-Zangbo suture zone to the south. Its collision with the Qiangtang terrane to the north during the Mesozoic marked the closure of the Meso-Tethyan Ocean, and subsequent collision with the Tethyan Himalaya to the south during the early Cenozoic marked the closure of the Neo-Tethyan Ocean. Therefore, the Mesozoic history of the Lhasa terrane provides key context for the Lhasa-Qiangtang collisional process, the amount of intracontinental shortening within Asia, as well as the shape of the southern margin of Asia before the India-Asia collision. However, previous palaeomagnetic results obtained from both volcanic and sedimentary rocks of Cretaceous age yielded discrepant palaeolatitudes ranging from 9°N to 26°N^9,10,13–25^. Thus, the palaeomagnetically constrained Lhasa-Qiangtang collisional ages range from the Middle Jurassic (~162 Ma)^26^ to the Late Cretaceous^27^, with the consequence that the estimated amount of post-collisional crustal shortening within Asia varies from >1800 km^5,19^ to only a few hundred kilometres^20^. Noticeably, the large palaeolatitude discrepancy may be attributed to several factors: (1) lower palaeolatitudes, observed from sedimentary rocks, may partly result from compaction-induced inclination shallowing^28^; (2) some volcanic palaeomagnetic datasets originated from analyses of too few lava flows to average palaeosecular variation^15,16^, or from stacks of lava flows whose attitudes were not accurately measured; (3) some palaeomagnetic datasets were from only a few sites that may be unreliable. The Lhasa terrane is long and narrow, with a length >2000 km from west to east and a width <300 km from north to south (Fig. 1a), yet most of the published palaeomagnetic data mainly come from the central part of the Lhasa terrane, while reliable palaeomagnetic datasets in its western part are relatively scarce. Therefore, high-quality Cretaceous palaeomagnetic data from the Lhasa terrane, especially from its far western part, are still necessary to better constrain the age of the Lhasa-Qiangtang collision, the amount of the intracontinental shortening within Asia, as well as the pre-collisional shape of the southern margin of Asia. In this study, we generated a new, high-quality and well-dated Early Cretaceous palaeomagnetic dataset from the Duoai Formation (Fm) lava flows and Jiega Fm limestone in the far western Lhasa terrane.

The Early Cretaceous Zenong Group volcano-sedimentary sequences cover an area of ~2.0 × 10^4^ km^2^ from Gar in the west to Nam Tso in the east and have an average thickness of more than 1000 m^29^. Our study area is located in the Shiquanhe area of the far western Lhasa terrane where the Zenong Group volcanic rocks and Jiega Fm limestone are extensively exposed (Fig. 1b). The Zenong Group strata consist of, from bottom to top, the Duoai, Tuocheng, and Langjiu Fms. The Duoai Fm is mainly composed of basalts and basaltic andesites. The Tuocheng Fm and Langjiu Fm primarily include dacites, tuffs or rhyolites, and trachytes or rhyolites, respectively. The zircon U-Pb ages of adjacent Zenong Group volcanic rocks in the Cuoqin area range from ~130 to 110 Ma^29^. Fossils identified in the Jiega Fm limestone include *Acanthochaetetes aff*. *seunesi Alloiteau*, *Orbitolina* sp., *Lucina* sp., *Ampullina xainzaensis Yu*, *Adiozoptyxis coquandiana*, *Pseudocucullase* sp., *and Freiastante* sp., and are indicative of the Early Cretaceous (1:250000 scale Shiquanhe regional geological survey report (I44C004002), 2004). The earliest folding of the Early Cretaceous strata in the study area likely occurred at the end (65–75 Ma) of the Late Cretaceous^2^.

A total of 301 oriented cores from 29 palaeomagnetic sites were collected from sections A and B (Fig. 1b). Twenty-three sites (ZN1–ZN23), encompassing 239 cores, were drilled from the Duoai Fm lava flows in section A (32°13.03′–32°13.05′N, 80°25.86′–80°25.92′E), located ~15 km south of the town of Zuozuo. The bedding attitudes could be exactly determined at the boundaries between two different lava flows or the intercalated sedimentary rocks (Supplementary Fig. S1a–f). Some lava flows displayed clear vesicular structures (Supplementary Fig. S1c). Although rhyolites of the Langjiu Fm are mapped as part of the Early Cretaceous Zenong Group (I44C004002), our sensitive high-resolution ion micro-probe (SHRIMP) zircon U-Pb age calculations indicate eruption at 22.6 ± 0.9 Ma and 24.2 ± 0.4 Ma^30^. We did not find any other Early Cretaceous volcanics with unambiguous, but distinctly different attitudes in the field area. We also collected 62 cores from six sites (ZZ1–ZZ6) in the Early Cretaceous Jiega Fm limestone in section B (32°9.3′–32°9.4′N, 80°47.7′–80°47.8′E), located ~40 km southeast of the town of Zuozuo, with the goal of performing a regional fold test (Supplementary Fig. S1g). Furthermore, two fresh block samples were collected from the bottom (ZN1) and top (ZN21) sites of section A for zircon U-Pb geochronology.

## Results

### U-Pb Zircon dating

**Figure 2:**
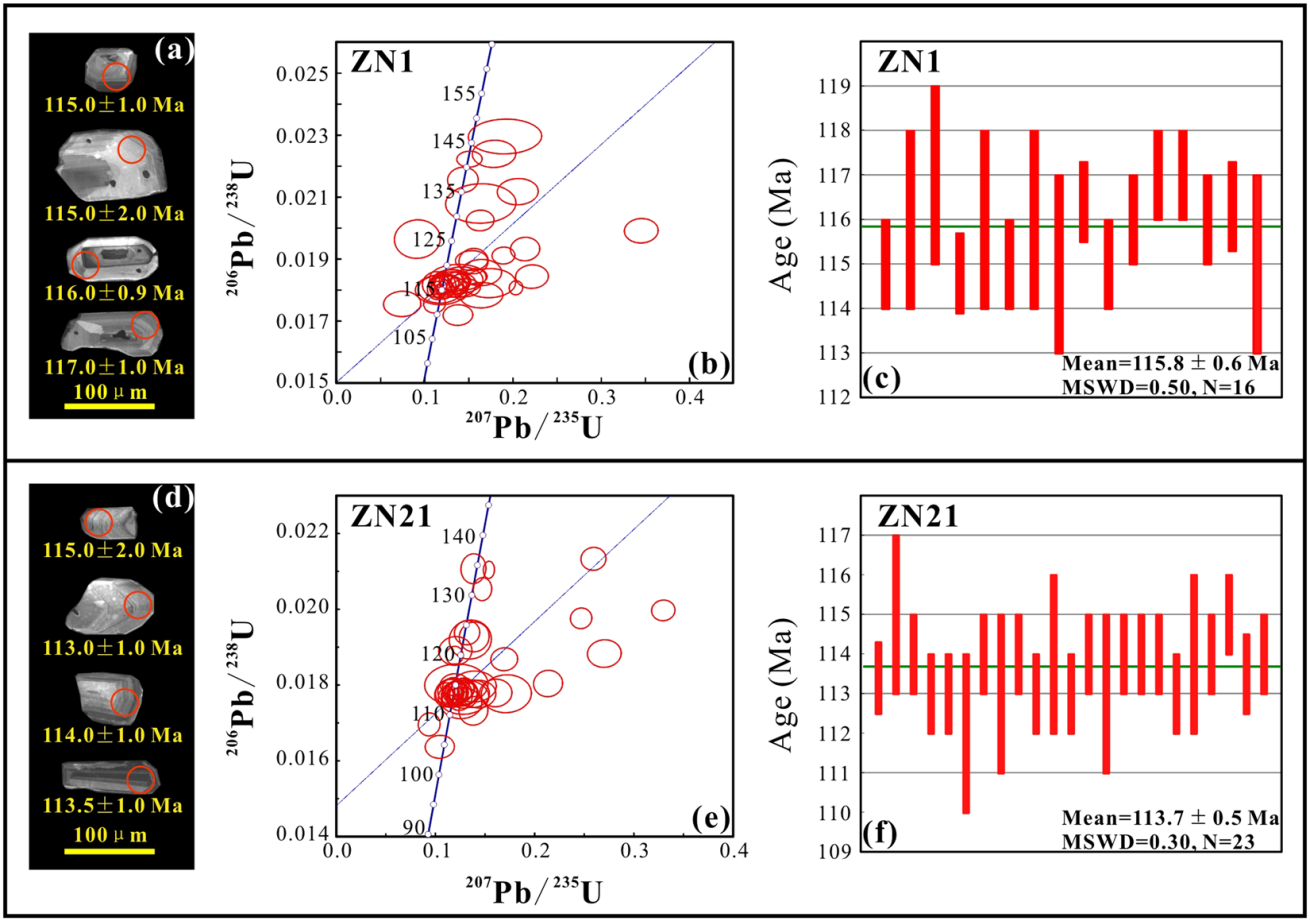
(**a**,**d**) Cathodoluminescence images of representative zircon grains from the samples ZN1 and ZN21, and corresponding ^206^Pb/^238^U ages of the individual analyzed spots. (**b**,**e**) U-Pb concordia diagrams of zircon grains; (**c**,**f**) bar plot shows the weighted mean ^206^Pb/^238^U ages.

Zircon crystals are euhedral to subhedral (Fig. 2a,d). These features, together with the clear oscillatory zoning observed in cathodoluminescence images, indicate that the zircons have a magmatic origin. Moreover, the U/Th ratio of the zircons from samples ZN1 (0.93−1.43) and ZN21 (0.87–1.81) were higher than metamorphic zircons (generally < 0.1). We interpret the weighted mean ^206^Pb/^238^U ages of the youngest age groups as the formation time of the volcanic rocks. The bottom (ZN1) and top (ZN21) samples of section A yielded weighted mean ^206^Pb/^238^U ages of 115.8 ± 0.6 Ma and 113.7 ± 0.5 Ma, respectively (Fig. 2), which indicate that the sampled Duoai Fm lava sequences erupted during the Early Cretaceous and were not overturned during folding.

### Rock magnetic and palaeomagnetic results

Isothermal remanent magnetisation (IRM) acquisition curves displayed a rapid increase below 200 mT, and complete saturation is often reached at less than 1500 mT (Supplementary Fig. S2a,d). When reverse fields were applied, the remanence decreased rapidly and then reduced to zero at 40–50 mT, revealing that low-coercivity magnetic minerals are dominant. Thermal demagnetisation of a three-axis IRM successively imparted at 2.4, 0.4, and 0.12 T on the mutually orthogonal sample axes^31^ indicated that the low-coercivity magnetic carriers unblocked at ~580 °C should be magnetite (Supplementary Fig. S2b,e). The single-cycle temperature dependence of magnetic susceptibility (χ-T curves) showed a decrease at ~580 °C (Supplementary Fig. S2c,f), revealing the presence of low-coercivity magnetite. The magnetic susceptibility increases substantially after heating, which may be attributed to newly formed magnetic minerals^11^ or to conversion of Ti-rich titanomagnetites to magnetite^32^. The slightly “pot-bellied” shape of the hysteresis loop also supports existence of low-coercivity magnetic minerals in the Duoai Fm lava samples (Supplementary Fig. S3a). On a Day plot^33,34^, these hysteresis parameters plot in a pseudo-single domain (PSD) region (Supplementary Fig. S3c). The PSD grains are very efficient carriers of remanent magnetisation, so they could have carried stable remanence when the Duoai Fm lava flows were erupted.

Scanning electron microscopy (SEM) and energy dispersive spectrometry (EDS) showed that Ti-Fe oxides are abundant in the studied Duoai Fm lava samples (Supplementary Fig. S4a,b,e–j). Backscattered electron (BSE) images revealed that the Ti-Fe oxide grains have a size range from several hundreds of nanometres to more than ten microns, and usually occur as irregular shapes distributed in silicates (Supplementary Fig. S4a,b). Furthermore, the BSE images showed that all of the Ti-Fe oxides grains have no obvious oxidised rims. These features, together with the rock magnetic results (Supplementary Figs S2 and S3), support the interpretation that magnetite formed by high-temperature exsolution during cooling, rather than from low-temperature transformation of original Fe oxides.

**Figure 3:**
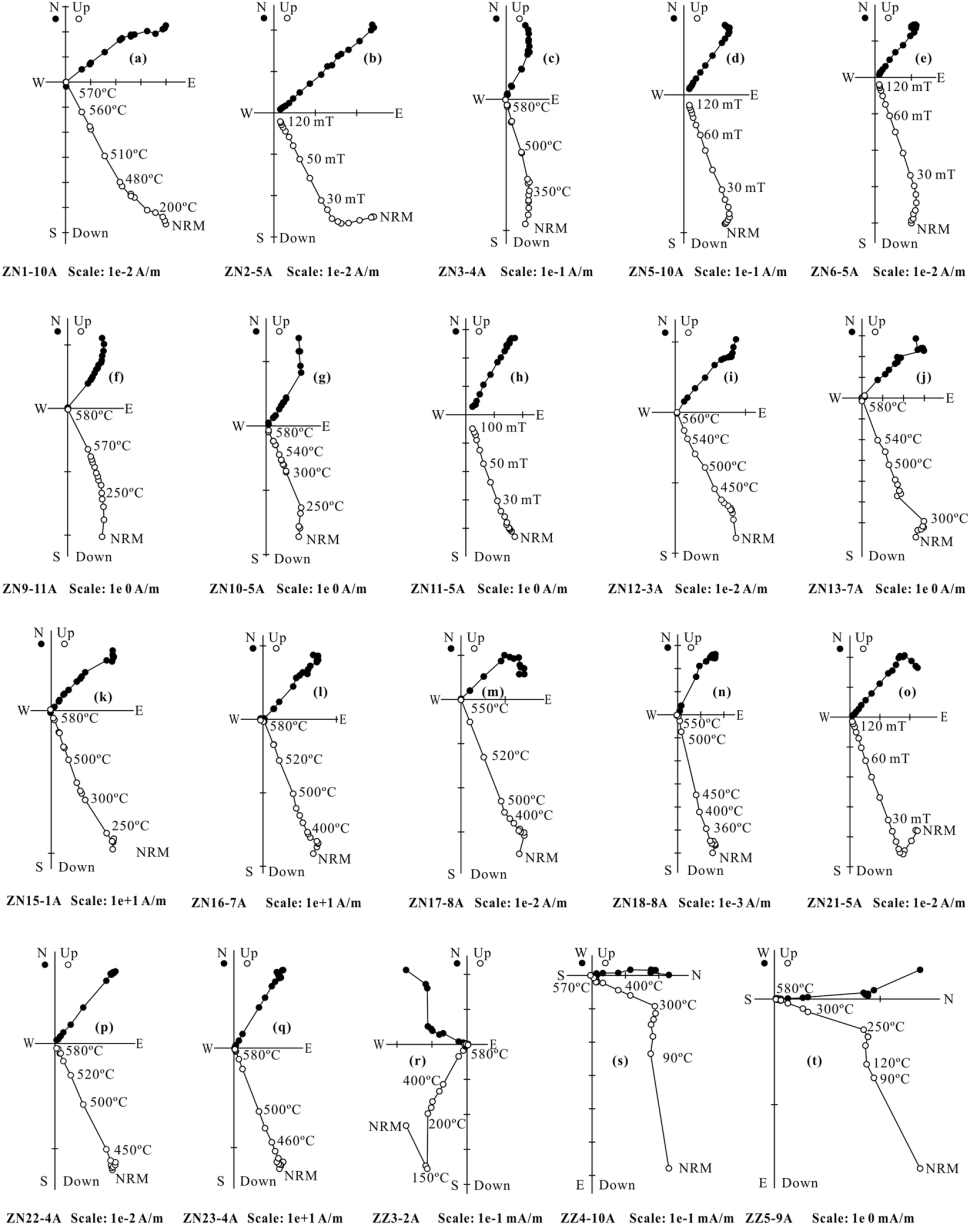
Thermal/alternating field demagnetization diagrams for representative specimens from the Duoai Fm lava flows (**a**–**q**) and the Jiega Fm limestone (**r**–**t**) in geographic coordinates. The solid and open symbols represent the projections onto the horizontal and vertical planes, respectively.

**Figure 4:**
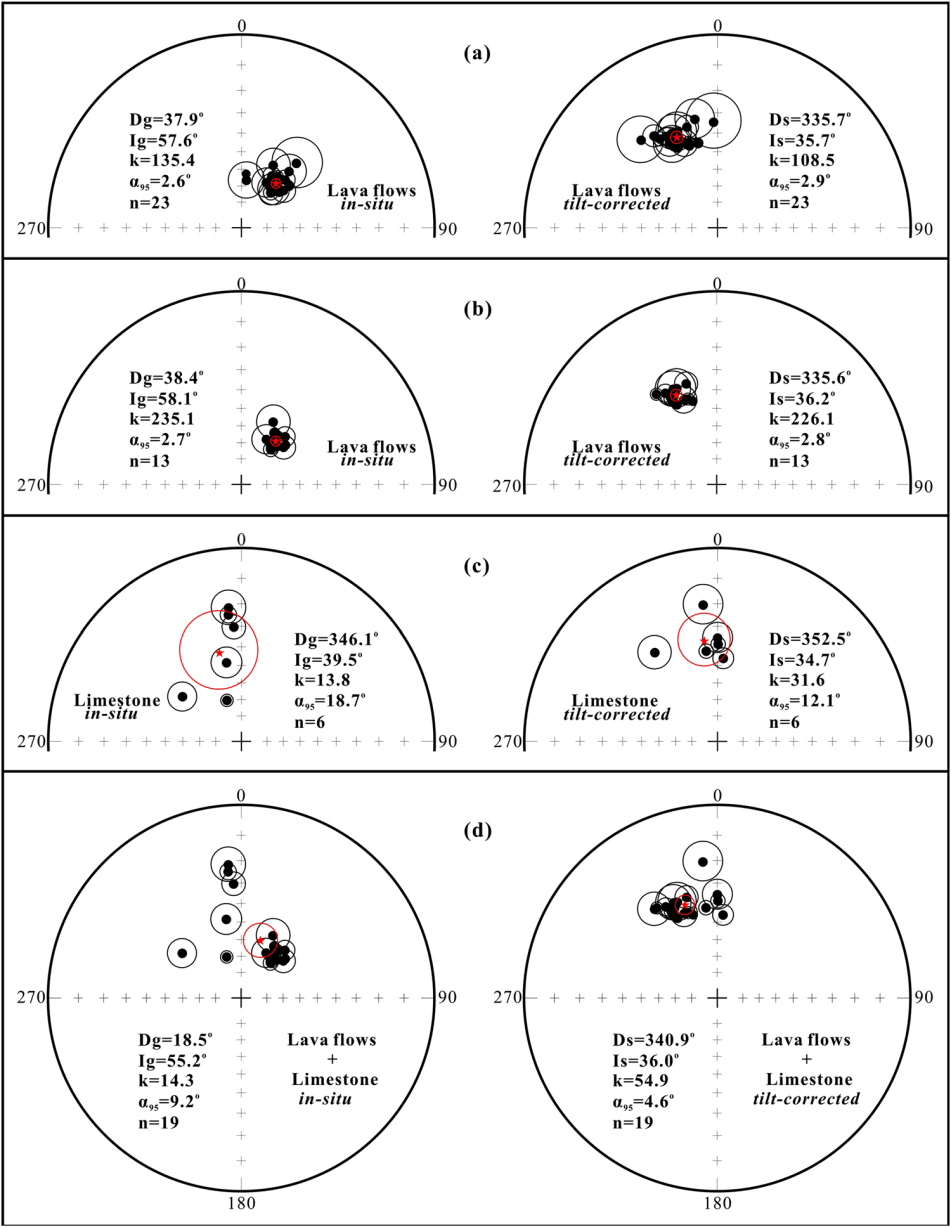
(**a**) Equal-area projections of site-mean directions from the Duoai Fm lava flows; (**b**) group-mean directions from the Duoai Fm lava flows; (**c**) group-mean directions from the Jiega Fm limestone; (**d**) group-mean directions from the Duoai Fm lava flows + Jiega Fm limestone. The stars indicate the Fisherian mean of site (group)-mean directions.

One hundred and seventy-seven samples underwent stepwise thermal demagnetisation, and 36 samples were treated with stepwise alternating field demagnetisation. Representative Zijderveld diagrams are shown in Fig. 3a–q. After removing a low-temperature component (LTC) or low-coercivity component, a stable univectorial high-temperature component (HTC) or high-coercivity component was isolated between 400 °C and 550–580 °C, or between 30 mT and 100–120 mT, and defines the characteristic remanent magnetisation (ChRM). An unblocking temperature of 550–580 °C, combined with the rock magnetic results, indicated that ChRM directions of the lava samples should be carried by magnetite. In addition, all the ChRM directions isolated from the Duoai Fm lava flows show a normal polarity, which is consistent with magnetisation acquired during the Cretaceous Long Normal Superchron^35^. Table 1 lists site-mean ChRM directions. The site-mean direction for the 23 Duoai Fm lava sites was Dg = 37.9°, Ig = 57.6°, kg = 135.4, α_95_ = 2.6° *in situ*, and Ds = 335.7°, Is = 35.7°, ks = 108.5, α_95_ = 2.9° after tilt-correction, corresponding to a palaeopole at 65.1°N, 326.6°E with A_95_ = 3.1° (Table 1 and Fig. 4a). The precision parameter (k-value) for the Duoai Fm lava flows decreased with unfolding. Because all of the Duoai Fm lava sites were collected entirely from a monoclinal section where bedding attitudes only have a very slight change (Table 1 and Supplementary Fig. S1), the decrease in the k-value of the Duoai Fm lava flows after unfolding can be attributed to the slightly biased tilt-correction, usually caused by local fluctuation of the bedding attitudes or measurement errors. Noticeably, when the ChRM directions of all the 23 Duoai Fm lava sites were corrected using the same average attitude (strike/dip = 206°/55°), they yielded a mean direction of Ds = 335.8°, Is = 35.1°, ks = 135.4, α_95_ = 2.6°. Obviously, this mean direction is very consistent with the site-mean direction (Ds = 335.7°, Is = 35.7°, ks = 108.5, α_95_ = 2.9°) of the 23 Duoai Fm lava sites corrected by the individual attitude, further suggesting that the k-value decrease of the Duoai Fm lava flows after unfolding is likely an artefact of field measurements and not partial pre-folding remagnetisation.

**Table 1:** Site (group)-mean ChRM directions of the Duoai Fm lava flows and Jiega Fm limestone from the Shiquanhe area in the far western Lhasa terrane.

Direction group	Site ID	Strike/Dip (°)	n/N (°)	Dg (°)	Ig (°)	Ds (°)	Is (°)	k (°)	α95 (°)	Plon (°)	Plat (°)
**Duoai Fm lava flows**											
D1	ZN1	209/51	10/10	47.8	58.1	341.1	41.8	699.7	1.8	329.2	71.5
D1	ZN2	209/51	9/9	48.8	53.7	347.1	41.6	469.7	2.4	317.6	76.0
D1	ZN1 + 2	209/51	19/19	48.3	56	343.9	41.7	405.8	1.7	324.4	73.6
D2	ZN3	209/51	7/10	29.0	60.2	335.4	33.1	55.8	8.1	323.8	63.7
	*ZN4	209/51	7/10	40.5	42.7	357.9	32.7	24.2	12.5	268.4	75.5
D3	ZN5	205/48	10/10	37.5	55.4	341.3	40.0	278.0	2.9	325.4	71.0
D4	ZN6	205/48	9/10	35.2	61.2	333.6	40.5	587.2	2.1	335.8	65.0
D5	ZN7	198/52	6/9	27.0	50.2	337.4	33.5	58.8	8.8	321.5	65.4
	*ZN8	198/52	7/10	6.0	62.3	318.9	28.0	36.8	10.1	335.4	48.9
D6	ZN9	196/63	9/10	35.1	54.2	327.0	32.6	254.7	3.2	332.5	57.0
D6	ZN10	196/63	8/8	30.4	55.5	325.3	30.0	131.3	4.9	331.6	54.7
D6	ZN9 + 10	196/63	17/18	32.9	54.8	326.2	31.4	176.8	2.7	332.0	55.9
D7	ZN11	205/54	10/10	31.4	57.9	334.1	33.1	702.3	1.8	325.4	62.7
D7	ZN12	205/54	10/10	40.3	57.7	335.6	37.7	718.7	1.8	329.4	65.6
D7	ZN11 + 12	205/54	20/20	35.9	57.9	334.8	35.4	448.2	1.5	327.3	64.1
D8	ZN13	205/54	10/10	42.0	60.6	332.1	38.9	410.2	2.4	334.9	63.2
	*ZN14	205/54	9/9	39.6	60.9	331.5	37.8	35.2	8.8	334.0	62.4
D8	ZN15	205/54	9/10	37.8	60.7	331.6	36.9	323.6	2.9	332.8	62.1
D8	ZN13 + 15	205/54	19/20	40	60.7	331.9	38.0	362.0	1.8	333.9	62.8
D9	ZN16	208/55	10/10	48.6	57.6	338.6	39.7	56.2	6.5	328.7	68.7
D10	ZN17	208/55	7/8	40.3	63.4	330.8	36.2	79.4	6.8	332.7	61.3
D10	ZN18	208/55	8/8	40.3	61.6	333.0	36.0	328.7	3.1	330.2	62.9
D10	ZN17 + 18	208/55	15/16	40.3	62.5	332.0	36.1	141.9	3.2	331.4	62.2
D11	ZN19	209/58	9/9	46.2	59.8	335.3	35.7	354.7	2.7	327.1	64.6
D11	ZN20	209/58	11/11	41.0	59.8	335.0	33.1	712.5	1.7	324.3	63.4
D11	ZN19 + 20	209/58	20/20	43.3	59.8	335.1	34.3	454.6	1.5	325.6	64.0
D12	ZN21	209/58	6/7	39.9	63.4	330.6	33.1	270.3	4.1	329.4	60.0
D13	ZN22	209/58	6/7	42.5	53.0	343.1	32.9	160.2	5.3	311.3	69.1
	*ZN23	209/58	8/8	40.3	48.3	348.2	30.4	40.4	8.8	297.2	70.9
**Sub-mean**	**(N = 23 sites)**			**37**.**9**	**57**.**6**	**335**.**7**	**35**.**7**	**108**.**5**	**2**.**9**	**326**.**6**	**65**.**1**
										**K = 98**.**5**	**A**_**95**_ ** = 3**.**1**
**Group mean**	**(N = 13 sites)**			**38**.**4**	**58**.**1**	**335**.**6**	**36**.**2**	**226**.**1**	**2**.**8**	**327**.**5**	**65**.**2**
										**K = 229**.**4**	**A**_**95**_ ** = 2**.**7**
**Jiega Fm limestone**											
T1	ZZ1	277.5/26.5	8/8	340.6	64.6	352.9	39.6	247.2	3.5	295.5	78.4
T2	ZZ2	277.5/26.5	5/9	349.3	45.0	354.0	19.4	97.5	7.8	276.1	67.1
T3	ZZ3	277.5/26.5	8/8	307.2	48.1	324.8	31.3	55.4	7.5	333.6	54.7
T4	ZZ4	48/17	8/8	354.1	23.4	0.4	36.6	263.3	3.4	259.0	78.2
T5	ZZ5	48/17	5/8	354.5	20.6	0.1	33.7	122.4	6.9	260.4	76.2
T6	ZZ6	54/18	7/9	356.2	28.8	4.0	43.4	137.0	5.2	232.9	82.3
**Group mean**	**(N = 6 sites)**			**346**.**1**	**39**.**5**	**352**.**5**	**34**.**7**	**31**.**6**	**12**.**1**	**289**.**6**	**75**.**6**
										**K = 31**.**5**	**A**_**95**_ ** = 12**.**1**
**Group mean**	**(N = 19 sites)**			**18**.**5**	**55**.**2**	**340**.**9**	**36**.**0**	**54**.**9**	**4**.**6**	**319**.**8**	**69**.**1**
										**K = 49**.**0**	**A**_**95**_ ** = 4**.**8**

*Notes:* Site ID, site identification; n/N, number of samples used to calculate mean and measured; Dg, Ig, Ds, and Is, declination and inclination in geographic and stratigraphic coordinates, respectively; k (K), the best estimate of the precision parameter; α_95_ (A_95_), the radius that the mean direction (pole) lies within the 95% confidence; Plat and Plon, latitude and longitude of palaeopoles in stratigraphic coordinates. *Sites were not used to calculate the final mean direction.

(1) The McFadden^37^ fold test for lava flows (N = 13) is positive at 95% confidence levels at “Xi2” test: critical Xi at 95% = 4.20. Xi2 IS = 5.00, Xi2 TC = 4.09.

(2) The McFadden^37^ fold test for limestones (N = 6) is positive at 95% and 99% confidence levels: critical Xi at 95% and 99% = 2.86 and 3.92. Xi1 and Xi2 IS = 4.40 and 5.17, Xi1 and Xi2 TC = 1.48 and 1.27, respectively.

(3) ① The McElhinny^43^ fold test for the lava flows and limestones (N = 19) is positive at 95% and 99% confidence levels: ks/kg = 3.84 > F(2*(n2-1), (n1-1)) at 5% and 1% point = 1.74 and 2.21, respectively; ② The McFadden^37^ fold test is positive at 99% confidence levels: critical Xi at 99% = 7.11. Xi1 and Xi2 IS = 15.39 and 17.27, Xi1 and Xi2 TC = 6.84 and 5.79, respectively.

The eruption of successive lavas can be emplaced faster than geomagnetic field changes, so instead of site-mean directions, group-mean directions for stratigraphically successive sites that share a common direction were calculated. Furthermore, we followed filtering criteria^36^ and excluded site and group mean directions with precision parameters (k-values) < 50 from further analysis. Nineteen of 23 palaeomagnetic sites were binned into 13 independent direction groups (Table 1 and Fig. 4b), which provided a group-mean direction of Dg = 38.4°, Ig = 58.1°, kg = 235.1, α_95_ = 2.7° *in situ*, and Ds = 335.6°, Is = 36.2°, ks = 226.1, α_95_ = 2.8° after tilt-correction, corresponding to a palaeopole at 65.2°N, 327.5°E with A_95_ = 2.7°. The decrease in the k-value for the 13 independent direction groups was less than the 23 site-mean directions after untilting, and the group-mean direction is fully consistent with the average of the site-mean directions (Table 1 and Fig. 4b). Furthermore, the group-mean direction passed the McFadden^37^ fold test at the 95% confidence level (Table 1), suggesting that the ChRMs obtained from the Duoai Fm lava flows record a pre-tilting magnetisation.

As palaeomagnetic directions from each lava flow provide only an instantaneous record of the geomagnetic field behaviour^38^, reliable palaeomagnetic data for geographical and tectonic reconstructions must average palaeosecular variations of the geomagnetic field. The A_95_ vale obtained from the virtual geomagnetic poles (VGPs) of 13 independent direction groups is 2.7°, consistent with the reliability envelope of A_95 min_/A_95 max_ at 1.6°/3.3° (N = 164)^39^. This, together with information from 13 independent direction groups from different lava flows interbedded with multiple layers of sedimentary rocks (Supplementary Fig. S1) spanning 113.7 ± 0.5 Ma to 115.8 ± 0.6 Ma (Fig. 2), supports the conclusion that the palaeomagnetic data obtained from the group-mean directions of the Early Cretaceous Duoai Fm lava flows have averaged palaeosecular variation.

### Jiega Fm limestone

IRM acquisition curves increased slowly below 1.2 T and rose rapidly above 1.2 T; saturation was not reached at 2.5 T (Supplementary Fig. S2g). When reverse fields were applied, the remanence decreased rapidly and reduced to zero after fields of 1.2–1.6 T, indicating that the Jiega Fm limestone samples contain high-coercivity magnetic minerals. The Lowrie^31^ test showed that the hard components were removed below 100 °C (Supplementary Fig. S2h), and thus we interpret the high-coercivity magnetic minerals to be goethite. Furthermore, the medium and soft components unblock at ~580 °C, which is consistent with the Curie point temperature of low-coercivity magnetite shown by single-cycle χ-T curves (Supplementary Fig. S2h,i). The “wasp-waisted” shape of the hysteresis loops also supports coexistence of low-coercivity and high-coercivity magnetic minerals in the Jiega Fm limestone (Supplementary Fig. S3b). On a Day plot^33,34^, these hysteresis data plot in a PSD region (Supplementary Fig. S3c) entirely within an area in which limestones carry primary remanence^40^.

BSE images and EDS analyses showed that the studied Jiega Fm limestone samples include abundant Fe oxides whose sizes ranged from several hundreds of nanometres to more than twenty microns (Supplementary Fig. S4c,d,k–m). We did not observe clusters of a few nanometre-sized grains common in remagnetised limestones^41,42^. In addition, neither obvious oxidised rims nor iron sulphides were observed in the Fe oxides. These features, combined with the rock magnetic results and the following palaeomagnetic results, support the interpretation that the Jiega Fm limestone likely retains a primary magnetisation.

As the studied Jiega Fm limestone samples contain abundant high-coercivity goethite, they were only demagnetised using stepwise thermal demagnetisation. Some samples recorded a LTC between 100 °C and 250–300 °C; these LTC directions in geographical coordinates are close to the present local geomagnetic field direction (Fig. 3r–t). After removing the LTC, a stable ChRM direction was isolated between 300 °C and 470–580 °C (Fig. 3r–t). An unblocking temperature of 470–580 °C, combined with the rock magnetic results (Supplementary Fig. S2g–i), indicates that the ChRM directions of Jiega Fm limestone samples are carried by magnetite. The site (group)-mean direction for the six limestone sites is Dg = 346.1°, Ig = 39.5°, kg = 13.8, and α_95_ = 18.7° *in situ* and Ds = 352.5°, Is = 34.7°, ks = 31.6, and α_95_ = 12.1° after tilt-correction, corresponding to a palaeopole at 75.6°N, 289.6°E with A_95_ = 12.1° (Table 1 and Fig. 4c). The site (group)-mean direction passes McFadden^37^ fold tests at the 95% and 99% confidence levels (Table 1), demonstrating that the ChRM directions obtained from the limestones were probably acquired before folding.

Significantly, the site-mean directions of the Jiega Fm limestone are consistent with those from the Duoai Fm lava flows, suggesting that the palaeomagnetic data obtained from the Jiega Fm limestone do not suffer from significant compaction-induced inclination shallowing^28^. Hence, the ChRM directions obtained from the Duoai Fm lava flows and Jiega Fm limestone can be averaged together, and they yielded an overall mean direction of Dg = 18.5°, Ig = 55.2°, kg = 14.3, α_95_ = 9.2° *in situ* and Ds = 340.9°, Is = 36.0°, ks = 54.9, α_95_ = 4.6° after tilt-correction (Fig. 4d). The overall mean direction passes both McElhinny^43^ and McFadden^37^ fold tests at 95% and 99% confidence levels (Table 1), indicating that the ChRMs should be primary magnetisation before folding. These 19 direction groups provide a Fisherian group-mean palaeopole at 69.1°N, 319.8°E with A_95_ = 4.8° (Table 1), corresponding to a palaeolatitude of 20.1° ± 4.8°N for the studied area (32.2°N, 80.4°E).

## Discussion

### Asian southern margin shape before the India-Asia collision

To appraise the reliability of paleomagnetic data, Van der Voo^44^ proposed seven quality criteria which include (1) well-determined rock age; (2) sufficient sample number (N > 24, k ≥ 10 and α_95_ ≤ 16.0); (3) proper demagnetization techniques; (4) structural control and tectonic coherence with the relevant craton or block involved; (5) reliable field tests; (6) the presence of reversals, and (7) no resemblance to paleopoles of younger ages (by more than a period). Because there exists a long normal polarity superchron (~126–83.6 Ma) during the Cretaceous^35^, we considered reliable Cretaceous palaeomagnetic data from the Lhasa and Qiangtang terranes those that satisfy all of the quality criteria (the 1–5 and 7) except for the criterion 6 mentioned above. Noticeably, the potential for inclination shallowing due to sediment deposition and compaction is still a critical and often unresolved problem for the accuracy of palaeomagnetic data from red beds^9,12,25^. However, we note that (1) available Cretaceous palaeomagnetic data of basalt flows intercalated with red beds indicated that the two site-mean ChRM directions for the interbedded red beds and basalt flows are consistent^25,45,46^; (2) the inclination-only mean of site-mean ChRM directions and its corresponding palaeolatitude for red beds compared with those for volcanic rocks showed that no significant compaction-induced inclination shallowing occurred in the Cretaceous red beds of the Lhasa terrane^9^; (3) only four Cretaceous palaeomagnetic datasets are available from the western Qiangtang terrane, and three of them were obtained from red beds; (4) although the elongation/inclination (E/I) method, which is based on a statistical model for palaeosecular variation of the geomagnetic field that satisfies a specific latitudinal dependence on a circular distribution of virtual geomagnetic poles^47^, has been widely used to check and correct sedimentary palaeomagnetic datasets with inclination shallowing, the foundation of the E/I method is still questionable because the latitudinal dependency of geomagnetic palaeosecular variation may be a mathematical artefact of the conversion from directions to poles^48^. In this study, we used sedimentary palaeomagnetic datasets without the E/I correction to calculate the palaeomagnetic poles (Table 2).

**Table 2:** Summary of Cretaceous palaeopoles from the Lhasa and west Qiangtang terranes, as well as from Eurasia and Mongolia.

ID	lithology	Area	Slat (°N)	Slon (°E)	Age (Ma)	Plat (°N)	Plon (°E)	A_95_ (dp/dm) (°)	Palaeolat (°N)	n/N	Criterion (Q)	References
**Cretaceous palaeomagnetic results from the western Lhasa terrane**												
CY	Limestone	Shiquanhe	32.7	80.2	K	67.7	234.2	13.1/24.5	11.8 ± 13.1	22/3	123γ5γ7 (5)	17
ZN	Volc	Cuoqin	31.4	85.1	~110–131	58.2	341.9	4.6	22.8 ± 4.6	162/18	123 F5R7 (7)	6
**SQ**	**Volc + Limestone**	**Shiquanhe**	**32**.**2**	**80**.**4**	**~113**–**116**	**69**.**1**	**319**.**8**	**4**.**8**	**20**.**1** ± **4**.**8**	**205/19**	**123F5**γ**7 (6)**	**This study**
QS	Volc	Yanhu	32.3	82.6	~120–132	61.4	192.9	2.1	18.2 ± 2.1	444/51	123F5D7 (7)	22
DZ	Volc	Cuoqin	31.1	84.4	~117–121	70.5	292.9	7.4	15.3 ± 7.4	116/12	123F*5D7 (7)	9
UC	Volc	Shiquanhe	32.4	80.1	~92.5	64.1	209.0	9.6	14.4 ± 9.6	78/10	123F5R7 (7)	23
LZ	Volc	Cuoqin	30.6	85.2	~99–93	63.1	224.6	5.1	9.5 ± 5.1	112/14	123F5γ7 (6)	21
LD	Volc	Shiquanhe	32.4	80.1	~68	47.7	180.3	3.4	17.2 ± 3.4	308/36	123F5D7 (7)	10
YR	Volc	Yare	31.6	82.2	~80	68.4	298.8	2.7	14.6 ± 2.7	136/15	123F5γ7 (6)	23
CQ	Redbeds	Cuoqin	31.2	84.7	K_2_	49.0	344.3	5.3	20.1 ± 5.3	291/33	123F5D7 (7)	9
**The mean palaeolatitude for the 100 reliable Early Cretaceous palaeomagnetic sites**									**18**.**8** ± **1**.**2**	**927/100**		**This study**
**The mean palaeolatitude for the 108 reliable Late Cretaceous palaeomagnetic sites**									**16**.**1** ± **1**.**4**	**925/108**		**This study**
**The mean palaeolatitude for the 208 reliable Cretaceous palaeomagnetic sites**									**17**.**4** ± **1**.**0**	**1852/208**		**This study**
**Cretaceous palaeomagnetic results from the central Lhasa terrane**												
DN	Volc + Sed	Naqu	31.3	91.9	~120.2	66.9	281.2	6.1	9.4 ± 6.1	139/19	123F5D7 (7)	24
WR	Volc	Deqing	30.5	90.1	~114	66.4	220.3	6.9	15.9 ± 6.9	88/15	123γγ5γ7 (5)	18
NQ	Volc	Naqu	31.5	92.0	~96	78.0	282.0	4.0/6.9	20.4 ± 4.0	33/9	123γγγ7 (4)	16
QL	Volc	Chalicuo	31.7	91.0	~90	74.0	318.0	11.1/19.1	20.7 ± 11.1	20/4	1γ3γγγγ (2)	16
CG	Volc + Redbeds	Linzhou	29.9	91.1	68–75	70.5	269.6	4.9	12.7 ± 4.9	164/21	123F5R7 (7)	25
TY	Redbeds	Dingqing	31.1	95.6	K_2_	71.4	273.1	5.2	13.6 ± 5.2	150/15	123F5γ7 (6)	12
MX	Redbeds + Volc	Maxiang	29.9	90.7	K_2_	75	306.7	6.8	19.7 ± 6.8	126/20	123F5γ7 (6)	19
SX	Volc	Linzhou	29.9	91.2	K_2_	69.1	191.7	3.3/5.4	26.2 ± 3.3	132/21	123F5γ7 (6)	20
TX	Redbeds	Linzhou	29.9	91.2	K_2_	70.2	300.5	1.4/2.7	14.6 ± 1.4	377/43	123F5D7 (7)	20
PJ	Redbeds	Linzhou	29.9	91.2	K_2_	68.0	340.0	6.7/11.6	22.3 ± 6.7	68/7	123F5γ7 (6)	13
WM	Redbeds	Linzhou	29.9	91.2	K_2_	64.0	348.0	5.6/9.5	23.3 ± 5.6	57/6	123F5γ7 (6)	14
AN	Redbeds	Barda	31.7	91.5	K_2_	63.5	325.4	6.5	14.9 ± 6.5	49/6	123F5γ7 (6)	15
AS	Redbeds	Linzhou	29.9	91.1	K_2_	71.2	288.4	7.9	14.1 ± 7.9	61/8	123F5γ7 (6)	15
**The mean palaeolatitude for the 166 reliable Cretaceous palaeomagnetic sites**									**16**.**1** ± **1**.**4**	**1323/166**		**This study**
**Cretaceous palaeomagnetic results from the western Qiangtang terrane**												
GZ1	Volc	Gaize	32.5	84.3	~104–111	79.3	339.8	5.7	29.7 ± 5.7	91/14	123F5γ7 (6)	27
LM	Redbeds	Longmucuo	34.5	80.4	Albian-Aptian	64.4	231.3	12.8	9.3 ± 12.8	41/4	123γ5D7 (6)	17
AK	Redbeds	Aksaichin	35.0	79.7	Albian-Aptian	66.3	256.5	6.6	8.5 ± 6.6	44/7	123F5γ7 (6)	17
GZ2	Redbeds	Gaize	32.5	84.3	K_2_-104	45.4	348.1	3.1	20.8 ± 3.1	174/22	123F5γ7 (6)	27
**The mean palaeolatitude for the 47 reliable Cretaceous palaeomagnetic sites**									**20**.**2** ± **3**.**5**	**350/47**		**This study**
**Eurasia**												
EU1					70	−79.2	355.7	2.5	30.6 ± 2.5			57
EU2					80	−79.7	357.9	2.9	30.3 ± 2.9			57
EU3					90	−80.4	347.2	2.5	32.2 ± 2.5			57
EU4					100	−80.8	332.3	3.3	34.6 ± 3.3			57
EU5					110	−81.2	13.1	3.3	35.2 ± 3.3			57
EU6					120	−79.0	10.1	2.6	27.9 ± 2.6			57
EU7					130	−75.0	3.4	2.8	27.7 ± 2.8			57
**EU**					**70~130**	**−79**.**6**	**357**.**8**	**2**.**4**	**30**.**3** ± **2**.**4**			**This study**
**Mongolia**												
MG					**92~125**	**80**.**8**	**158**.**4**	**2**.**5**	**33**.**7** ± **2**.**5**			58

*Notes:* ID, palaeopoles abbreviation used in the plot and text; volc, volcanic rocks; sed, sedimentary rocks; K, Cretaceous; K_2_, Late Cretaceous; Slat (Slon), latitude (longitude) of sites; Plat (Plon), latitude (longitude) of poles; A_95_, the radius that the mean pole lies within 95% confidence; dp/dm, semi-axes of elliptical error of the pole at a probability of 95%; Paleolat, palaeolatitude calculated for the reference point at (32.2°N, 80.4°E) for the western Lhasa terrane, western Qiangtang terrane, Eurasia and Mongolia; at (32.2°N, 91.1°E) for the central Lhasa terrane; n/N, number of samples or sites (groups) used to calculate Fisher mean; Criteria (Q), data quality criteria (number of criteria met) after Van der Voo^44^ [1, well determined rock age; 2, sufficient sample number (N > 24, k ≥ 10 and α_95_ ≤ 16.0); 3, proper demagnetization techniques; 4, field tests; 5, structural control and tectonic coherence with the craton or block involved; 6, the presence of reversals; 7, no resemblance to paleopoles of younger ages (by more than a period); F, positive fold test; R, positive reversal test; D, dual-polarity; “γ”, failed to meet this criterion].

**Figure 5:**
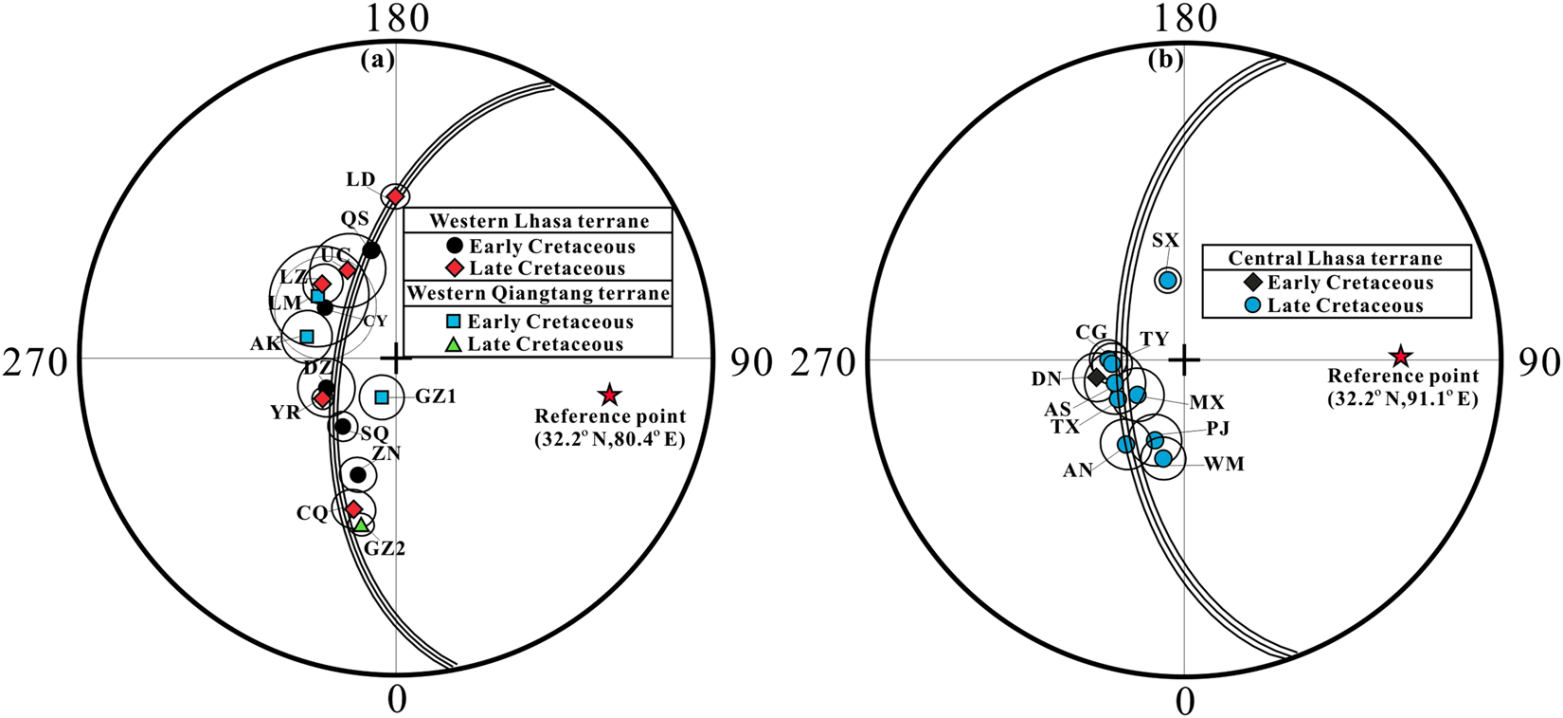
(**a**) Equal-area projections showing the Cretaceous palaeomagneitc poles obtained from the western Lhasa and Qiangtang terranes; (**b**) from the central Lhasa terrane. See Table 2 for the abbreviation and values. The small circle with its 95% confidences traversing a palaeolatitude of 17.4° ± 1.0°N calculated from the 208 Cretaceous palaeomagnetic sites of the western Lhasa terrane and 16.1° ± 1.4°N calculated from the 166 Cretaceous palaeomagnetic sites of the central Lhasa terrane.

The pre-collisional shape of the southern margin of Asia is a key constraint for estimating the diachroneity of the India-Asia collision. For the western Lhasa terrane (west of 87°E), a total of 10 Cretaceous palaeopoles have been published (Table 2 and Fig. 5a); their palaeolatitudes have been calculated for a reference point in our study area (32.2°N, 80.4°E). Notably, one Cretaceous palaeopole (CY)^17^ did not meet our selection criteria because it was calculated from only three sites (22 samples) and lacked a robust field test. We thus excluded this pole from further analysis. The other nine palaeopoles, which include four Early Cretaceous (ZN, SQ, QS, and DZ) and five Late Cretaceous (UC, LZ, LD, YR, and CQ) poles, fulfilled our quality criteria (Table 2). Among them, the four Early Cretaceous palaeopoles yielded palaeolatitudes of 20.1° ± 4.8°N (Shiquanhe area, this study), 22.8° ± 4.6°N and 15.3° ± 7.4°N (Cuoqin area)^6,9^, and 18.2° ± 2.1°N (Yanhu area)^22^. These four palaeolatitudes are consistent within 95% confidence limits, and their palaeopoles generally fall along a small circle centred at the reference location (Fig. 5a). This suggests that local vertical axis rotations have occurred within the western Lhasa terrane since the Early Cretaceous. We therefore follow Lippert *et al*.^36^ and follow the method of Doubrovine and Tarduno^49^ with the procedure of Arason and Levi^50^ to obtain an inclination-only mean of 34.2° ± 1.8° and a corresponding palaeolatitude of 18.8° ± 1.2°N for 100 reliable Early Cretaceous palaeomagnetic sites (Supplementary Table S1). The five Late Cretaceous palaeopoles from the western Lhasa terrane provide palaeolatitudes of 14.4° ± 9.6°N and 17.2° ± 3.4°N (Shiquanhe area)^10,23^, 20.1° ± 5.3°N and 9.5° ± 5.1°N (Cuoqin area)^9,21^, and 14.6° ± 2.7°N (Yare area)^23^. Considering that these five Late Cretaceous palaeopoles are statistically indistinguishable within 95% confidence limits and lie along a small circle centred on the reference location (Fig. 5a), we calculated an inclination-only mean of 30.0° ± 2.2° and a corresponding palaeolatitude of 16.1° ± 1.4°N for 108 reliable Late Cretaceous palaeomagnetic sites (Supplementary Table S1). This inclination and palaeolatitude is similar to (or slightly lower than) the 34.2° ± 1.8° and 18.8° ± 1.2°N calculated from the 100 reliable Early Cretaceous palaeomagnetic sites. These palaeomagnetic results support the interpretation that the western Lhasa terrane maintained a relatively stable palaeolatitude during the Cretaceous.

For the central Lhasa terrane, a total of 13 Cretaceous palaeopoles have been published (Table 2). We used a reference point of 91.1°E longitude with the same latitude (32.2°N) as the western reference point to calculate predicted palaeolatitudes for the central Lhasa terrane. It is worth noting that one Early Cretaceous palaeopole (WR)^18^ and two Late Cretaceous palaeopoles (NQ and QL)^16^ did not meet our selection criteria due to a lack of robust field tests and/or absence of a structural control; we thus excluded these three poles from further analysis. We calculated an inclination-only mean of 30.0° ± 2.2° and a corresponding palaeolatitude of 16.1° ± 1.4°N for 166 reliable Cretaceous palaeomagnetic sites from the central Lhasa terrane (Supplementary Table S1). These results are indistinguishable from the inclination-only mean of 32.1° ± 1.5° and the corresponding palaeolatitude of 17.4° ± 1.0°N for 208 reliable Cretaceous palaeomagnetic sites in the western Lhasa terrane (Supplementary Table S1). Thus, our analysis utilizing a statistically consistent framework and high-quality observations from western Tibet support earlier inferences that the pre-collisional southern margin of Asia had a relatively east-west alignment^5,22,51^. Our work is distinct from these earlier studies for its analytical approach and higher palaeolatitude estimates.

### The age of the Lhasa-Qiangtang collision

**Figure 6:**
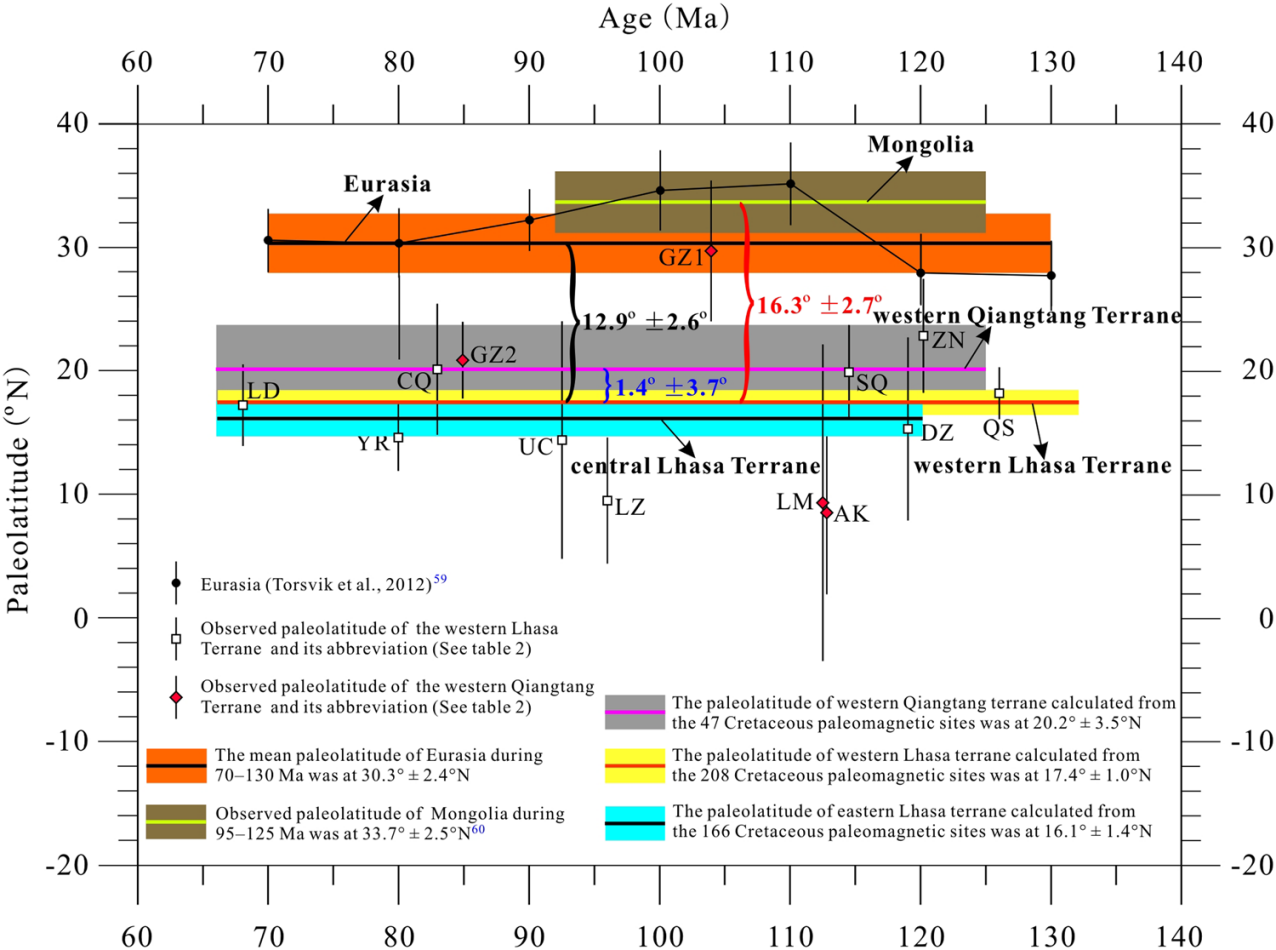
Palaeolatitude plots for Mongolia, Eurasia, the Lhasa and western Qiangtang terranes. The palaeolatitudes of Mongolia, Eurasia, the western Lhasa and Qiangtang terranes were calculated using the reference point (32.2°N, 80.4°E) in our study area, and reference point (32.2°N, 91.1°E) for the central Lhasa terrane.

Convergence between Lhasa and Qiangtang was essentially north to south, and thus the age of their collision can be determined by the palaeolatitude overlap of these two terranes at the same reference point. Because the Lhasa**-**Qiangtang collision may have been diachronous, we only considered the western part of this collision zone (west of 87°E). We note that no reliable Late Jurassic palaeomagnetic poles are available in the western Lhasa and Qiangtang terranes, and that the Qiangtang terrane has been located along the southern margin of the Palaeo-Asian continent since the Late Triassic closure of the Palaeo-Tethys Ocean^52^. Therefore, we first tested whether the Lhasa terrane overlapped with the Qiangtang terrane during the Early Cretaceous. All four of the Cretaceous palaeomagnetic poles obtained from the western Qiangtang terrane satisfied the selection criteria (Table 2) and provide palaeolatitudes of 8.5° ± 6.6°N (Aksaichin area)^17^, 9.3° ± 12.8°N (Longmuco area)^17^, and 20.8° ± 3.1°N and 29.7° ± 5.7°N (Gaize area)^27^. Considering that these four poles have a wide palaeolatitude range and fall along a small circle centred in the reference location (Fig. 5a), we also calculated an inclination-only mean of 36.4° ± 5.1° and a corresponding palaeolatitude of 20.2° ± 3.5°N for all of the 47 Cretaceous palaeomagnetic sites. Comparing this palaeolatitude with the Early Cretaceous palaeolatitude of 18.8° ± 1.2°N observed from the western Lhasa terrane shows an insignificant palaeolatitude difference of 1.4° ± 3.7°, and thus clear overlap between the two terranes. These results also correspond to a north-south crustal shortening of 150 ± 410 km. Although the ~1.4° (~150 km) crustal shortening is significantly less than the palaeomagnetic confidence limit (~3.7° or ~400 km) and the geological limit of palaeomagnetic studies (±500 km), the most likely shortening (~150 km) deduced from the palaeomagnetic results is consistent with the 120–250 km absorbed by the Cenozoic Shiquanhe-Gaize-Amdo thrust system^1^. This observation, combined with the conclusion that western Lhasa terrane maintained a relatively stable palaeolatitude throughout the Cretaceous (Fig. 6), suggests that the western Lhasa and Qiangtang terranes had collided by the Early Cretaceous. A recent report of a well-dated palaeomagnetic pole (51.7°N, 305.8°E with dp/dm = 1.7°/3.4°) from ~180 Ma volcanic rocks of the central Lhasa terrane yielded a palaeolatitude of 2.9 ± 1.7°N for the western reference point (32.2°N, 80.4°E)^11^. If we use the palaeolatitude (20.2 ± 3.5°N) of the western Qiangtang terrane as the southern margin of Palaeo-Asia before the Lhasa-Qiangtang collision and assume that the Lhasa terrane had an average northward velocity of 5 cm/yr between ~220 and ~130 Ma^11^, then the Lhasa terrane would intersect with the Qiangtang terrane at ~142 Ma, consistent with the geological interpretation that the Lhasa and Qiangtang terranes had collided by the Early Cretaceous. Geological evidence suggesting collision includes: (1) remnants of a Late Jurassic–Early Cretaceous subduction-accretion complex and forearc basin consisting of ophiolitic melange structurally overlain by Jurassic flysch near the Shiquanhe area^2^; (2) structural relationships and geochronological studies in the Nima basin along the Bangong-Nujiang suture zone showed that this basin underwent major structural deformation and erosion from a switch from marine to non-marine conditions between ~125 Ma and ~118 Ma, which are related to the Lhasa-Qiangtang terranes colliding during the Early Cretaceous^53^; (3) structural mapping and detrital zircon U-Pb dating in Domar from the western Qiangtang terrane that indicate significant shortening during Late Jurassic–Early Cretaceous, rather than the Cenozoic, in response to the India-Asia collision^54^; (4) synthesis of lithostratigraphical, magmatic, and metamorphic results from the Lhasa-Qiangtang collision zone that support a Bangong-Nujiang Ocean closure at ~130–140 Ma^55^; and (5) U-Pb and Hf isotopic analysis of detrital zircons from sedimentary rocks in the Gaize area that suggest the 140–130 Ma magmatic gap may represent the Lhasa-Qiangtang collision^56^.

### The magnitude of intracontinental shortening within Asia

The intracontinental shortening between the Lhasa terrane and stable Asia can be estimated by comparing palaeolatitudes from the same reference point with palaeolatitudes calculated from various reference poles. We employed two methods for selecting reference poles to constrain the intracontinental shortening. First, we compared our results with the Cretaceous (70–130 Ma) mean pole from the Eurasian apparent polar wander paths^57^. Second, we compared our results directly to a large dataset obtained from coeval lavas in Mongolia^58^, similar to previous approaches^36^. Reference poles for Mongolia and Eurasia are shown in Table 2. Considering that the western Lhasa terrane had already accreted to the stable Asian continent by the Early Cretaceous, as well as maintained a relatively stable palaeolatitude during the entire Cretaceous, we used the palaeolatitude of 17.4° ± 1.0°N, calculated from the inclination-only mean of 208 Cretaceous palaeomagnetic sites, as the palaeolatitude of the western Lhasa terrane before the India-Asia collision (Fig. 6). Comparing this latitude with 30.3° ± 2.4°N expected from the average pole of Eurasia from 70–130 Ma and 33.7° ± 2.5°N observed from the mean pole of Mongolia from 92–125 Ma reveals a palaeolatitudinal difference of 12.9° ± 2.6° and 16.3° ± 2.7° for the reference point (32.2°N, 80.4°E). This comparison implies a latitudinal convergence of ~1400 ± 290 km and ~1800 ± 300 km has occurred between the western Lhasa terrane and stable Asia and between the western Lhasa terrane and Mongolia, respectively, since the Early Cretaceous.

The discrepancies between the Mongolian and Eurasian reference poles could from a number of factors^36,59^. We conclude that the reference poles for Mongolia are the best determined palaeopoles for stable Asia. The ~1800 km north-south shortening deduced from comparison with reliable Cretaceous palaeomagnetic data from Mongolia is compatible with previously published palaeomagnetic estimates of 1700–2000 km since the end of the Cretaceous^19^.

## Conclusions

We have presented geochronological and palaeomagnetic results from the Early Cretaceous Duoai Fm lava flows and Jiega Fm limestone in the western Lhasa terrane. These new data, as well as previous Cretaceous palaeomagnetic data from the Lhasa and western Qiangtang terranes, Eurasia, and Mongolia, led us to reach the following conclusions: (1) the Duoai Fm lava flows of the Shiquanhe area erupted at ~113–116 Ma, consistent with the adjacent Zenong Group volcanic rocks in the Cuoqin area; (2) 19 direction groups from the Duoai Fm lava flows and Jiega Fm limestone provide a high-quality Early Cretaceous pole located at 69.1°N, 319.8°E (A_95_ = 4.8°), corresponding to a palaeolatitude of 20.1° ± 4.8°N for the Shiquanhe area; (3) consistent inclinations measured in the Early Cretaceous Duoai Fm lava flows and Jiega Fm limestone indicate that compaction-induced inclination shallowing is insignificant in the Jiega Fm limestone; (4) the shape of the southern margin of Asia had a relatively east-west alignment before the India-Asia collision; (5) the western Lhasa terrane had accreted to the stable Palaeo-Asian continent by the Early Cretaceous and maintained a relatively stable palaeolatitude during the entire Cretaceous; (6) comparison of the Cretaceous palaeolatitudes calculated from the western Lhasa terrane with a coeval reference pole for Mongolia reveals a latitudinal convergence of ~1800 ± 300 km.

## Methods

All of the samples were collected in the field using a portable gasoline-powered drill. Palaeomagnetic cores were oriented using both a magnetic compass and a sun compass to evaluate any discrepancies between local declinations and the present geomagnetic field in the study area. The differences between the magnetic and sun compass readings are less than 3°, indicating that the local magnetic disturbance can be neglected for all samples.

The standard oriented cores (2.5 cm diameter) were cut into 2.2-cm-long samples in the laboratory. Remanent magnetisation was measured using a JR-6A spinner magnetometer or a 2G 755-4 K cryogenic magnetometer, and demagnetisation was carried out with an ASC-TD 48 furnace with internal residual field lower than 10 nT or with a D-2000 alternating field demagnetiser. In principle, demagnetisation temperature intervals are 50 °C in the low temperature range and 30–10 °C in the high temperature range. Progressive alternating field demagnetisation was performed from 2.5 mT up to 100–120 mT. The instruments of the ASC-TD 48 furnace and magnetometers are housed in a magnetically shielded room with a magnetic field less than 300 nT. All of these experiments were completed at the Paleomagnetism and Environmental Magnetism Laboratory (PEML) at the China University of Geosciences, Beijing (CUGB). ChRM directions were obtained using principal component analysis of at least five successive steps^60^. Site-mean directions were analysed using Fisher statistics^61^. Published computer program packages^62,63^ were used to analyse palaeomagnetic data.

With the goal of identifying magnetic carriers and guaranteeing the reliability of palaeomagnetic data, the acquisition of IRM, back-field demagnetisation of saturation IRM, and thermal demagnetisation of three-axis IRM, hysteresis loops were measured for some representative samples. The acquisition of IRM, back-field demagnetisation of saturation IRM, and thermal demagnetisation of three-axis IRM were performed using an ASC IM10–30 pulse magnetiser and were measured with a JR-6A spinner magnetometer. These experiments were also completed at the PEML. The hysteresis loops were measured at room temperature using a MicroMag™ Model 3900 Vibrating Sample Magnetometer at the Paleomagnetism and Geochronology Laboratory at the Institute of Geology and Geophysics (Chinese Academy of Sciences).

SEM observations were performed with an acceleration voltage of 15 kV at the State Key Laboratory of Tribology (Tsinghua University, Beijing). The polished samples were coated with carbon before analysis to yield high-quality images. Energy dispersive spectrometry (EDS) was subsequently performed to acquire compositional information.

Zircons were obtained from bulk samples after they were crushed and ground, and separated by a combination of heavy liquids and magnetic techniques at the Langfang Laboratory of Geophysical Exploration (Geological Exploration Bureau of Hebei Province, Ministry of Land and Mineral Resources). Individual zircons were hand-picked under a binocular microscope, mounted in epoxy resin and polished to clearly expose the grain structure. Cathodoluminescence images were produced to reveal internal structures and choose potential target sites for U-Pb isotopic analyses. U-Pb zircon dating was conducted by laser ablation multicollector inductively coupled plasma mass spectrometry (LA-ICP-MS) at the Key Laboratory of Continental Collision and Plateau Uplift (Institute of Tibetan Plateau Research, Chinese Academy of Sciences, Beijing). To eliminate contamination, each zircon surface was cleaned with ethanol before analysis. U-Th-Pb concentrations were calibrated using National Institute of Standards and Technology (NIST) SRM 612 as an external standard and ^29^Si as an internal standard. More detailed procedures of the analytical techniques and the configuration of the LA-ICP-MS system are found in Cai *et al*.^64^.

## Electronic supplementary material

Supplementary Information
