# Supplementary material for: New Early Cretaceous palaeomagnetic and geochronological results from the far western Lhasa terrane: Contributions to the Lhasa-Qiangtang collision
Source: Sci Rep. 2017 Nov 24;7:16216. doi: 10.1038/s41598-017-16482-3 (PMC5701245; doi:10.1038/s41598-017-16482-3)
Supplement: Supplementary file 1 — Supplementary Information [file 41598_2017_16482_MOESM1_ESM.pdf]

Supplementary Information

New Early Cretaceous paleaomagnetic and geochronological results from the far western Lhasa terrane: Contributions to the Lhasa–Qiangtang collision

Weiwei Bian[a,b], Tianshui Yang[a,b*], Yiming Ma[a,b,c], Jingjie Jin[a,b], Feng Gao[a,b], Shihong Zhang[a,b], Huaichun Wu[a], Haiyan Li[a]

Supplementary Figure S1

Supplementary Figure S2

Supplementary Figure S3

Supplementary Figure S4

Supplementary Table S1

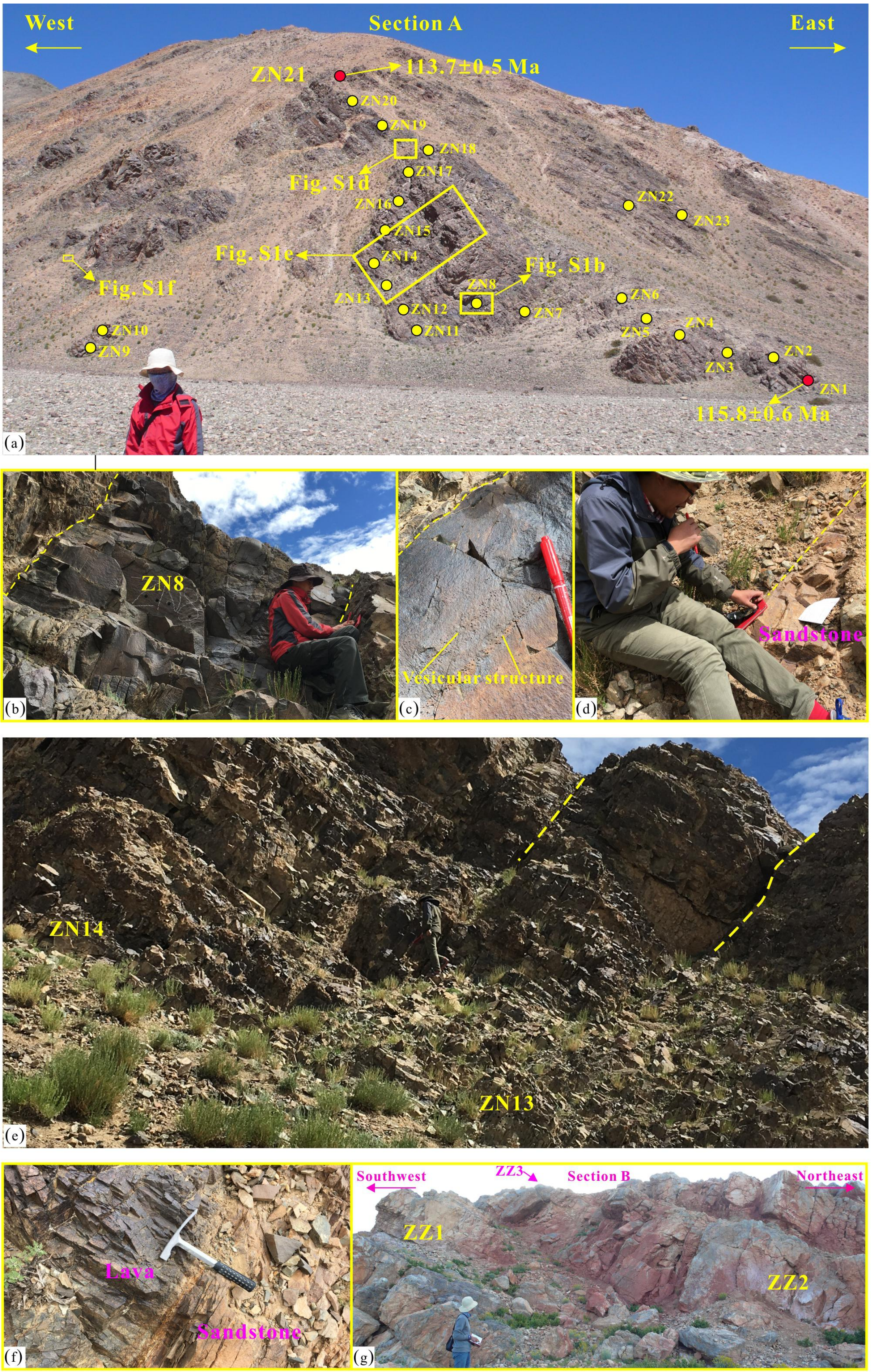

Supplementary Figure S1. Photographs showing the field outcrops of the Early Cretaceous Duoai Fm lava flows (a-f) and Jiega Fm limestone (g), respectively.

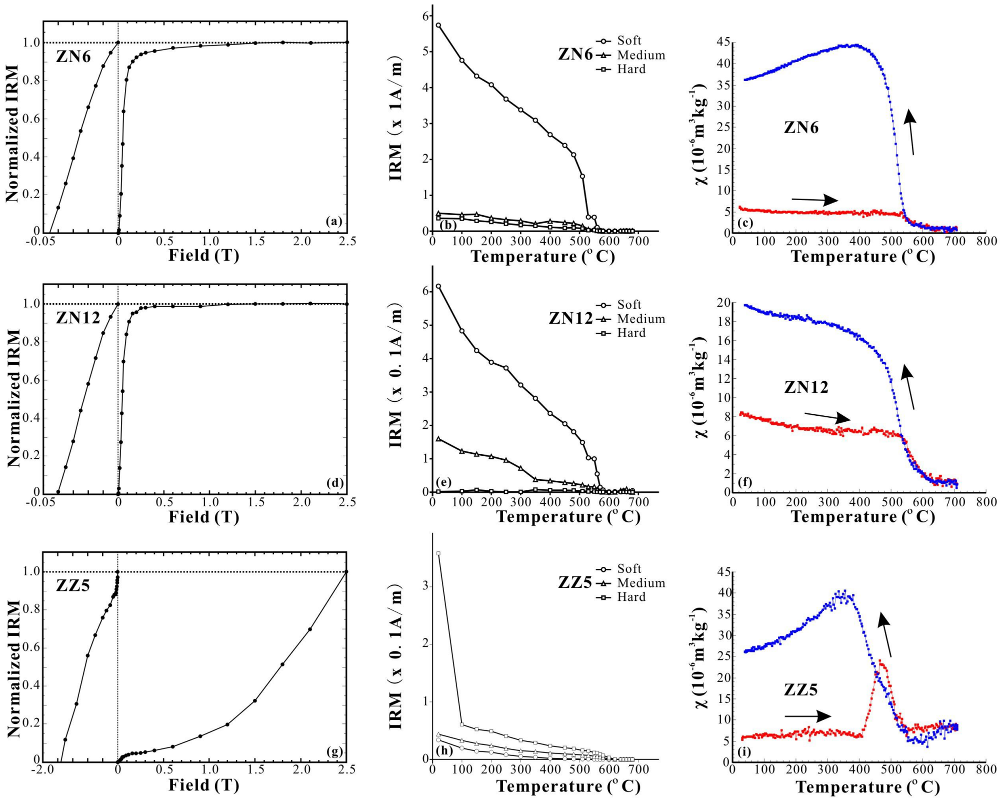

Supplementary Figure S2. Isothermal remanent magnetization (IRM) acquision curves and back–field demagnetization of saturation IRM cures (SIRM) for the Duoai Fm lava flows (a,d) and Jiega Fm limestone (g); Thermal demagnetization of three-axis IRM for the Duoai Fm lava flows (b,e) and Jiega Fm limestone (h) by magnetizing the specimens in 2.4 T along the z-axis, followed by 0.4 T along the y-axis, and finally 0.12 T along the x-axis; Temperature-dependence of magnetic susceptibility curves for the Duoai Fm lava flows (c,f) and Jiega Fm limestone (i).

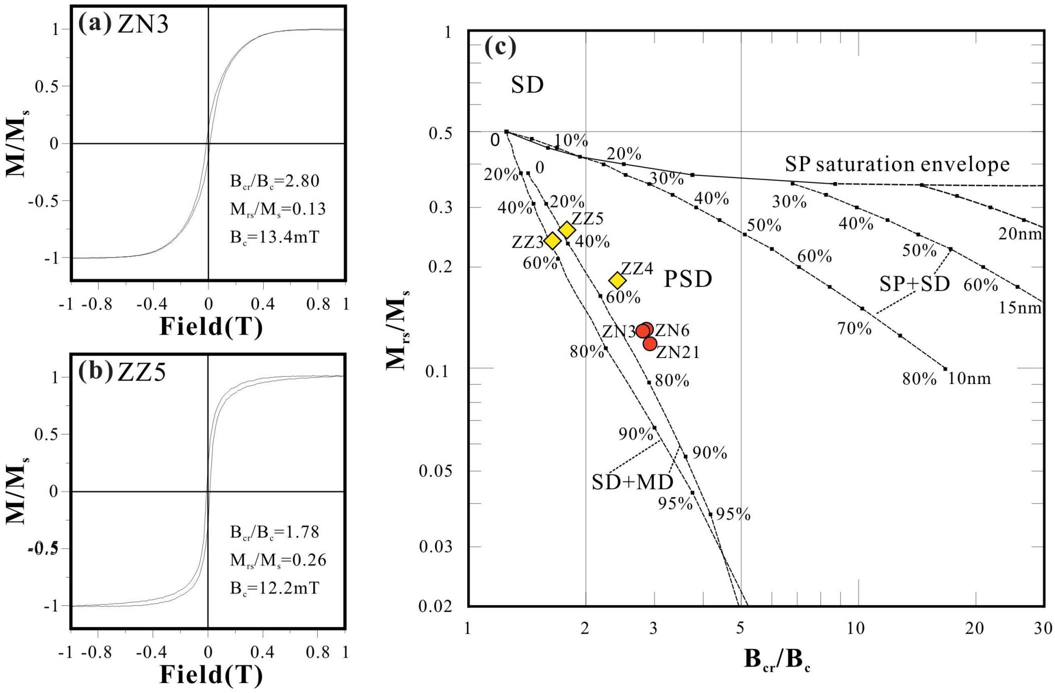

Supplementary Figure S3. Hysteresis loops for the (a) Duoai Fm lava flows and (b) Jiega Fm limestone; (c) Day plot[33,34] of the hysteresis parameters for the representative samples.

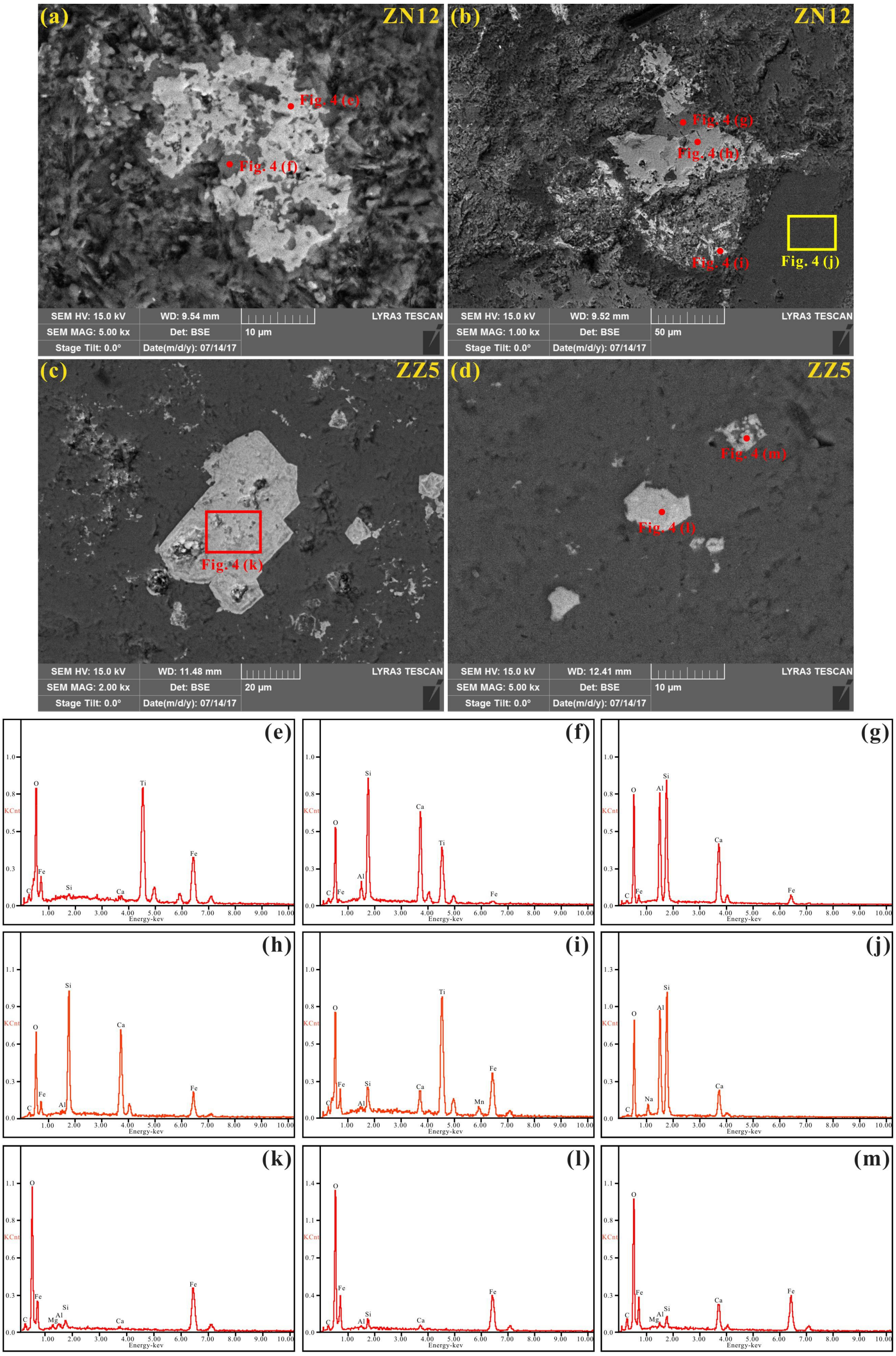

Supplementary Figure S4. SEM backscattered electron images (a-d) and EDS analyses (e-m) of representative samples from Duoai Fm lava flows and Jiega Fm limestone. Boxes and solid circles in the graphs show the spots analyzed by EDS.

Supplementary Table S1. Summary of the available Cretaceous palaeomagnetic results from the Lhasa and Qiangtang terranes.

Site	Plat(°N)	Plon(°E)	Rlat(°N)	Rlon(°E)	Dr(°)	Ir(°)
**The western Lhasa terrane**						
This study						
The Early Cretaceous (~113–116 Ma) Zenong Gp volcanic rocks and Jiega Fm limestone results						
D1	73.6	324.4	32.2	80.4	343.9	41.7
D2	63.7	323.8	32.2	80.4	335.4	33.1
D3	71.0	325.4	32.2	80.4	341.3	40.0
D4	65.0	335.8	32.2	80.4	333.6	40.5
D5	65.4	321.5	32.2	80.4	337.4	33.5
D6	55.9	332.0	32.2	80.4	326.2	31.4
D7	64.1	327.3	32.2	80.4	334.8	35.4
D8	62.8	333.9	32.2	80.4	331.9	38.0
D9	68.7	328.7	32.2	80.4	338.6	39.7
D10	62.2	331.4	32.2	80.4	332.0	36.1
D11	64.0	325.6	32.2	80.4	335.1	34.3
D12	60.0	329.4	32.2	80.4	330.6	33.1
D13	69.1	311.3	32.2	80.4	343.1	32.9
T1	78.4	295.5	32.2	80.4	352.8	39.6
T2	67.1	276.1	32.2	80.4	353.9	19.5
T3	54.7	333.6	32.2	80.4	324.6	31.6
T4	78.2	259.0	32.2	80.4	0.3	36.6
T5	76.2	260.4	32.2	80.4	360.0	33.6
T6	82.3	232.9	32.2	80.4	3.9	43.4
Previous studies						
The Early Cretaceous (~120–132 Ma) Qushenla Fm volcanic results from Yanhu area reported by Ma et al.[22]						
YH1	76.9	206.8	32.2	80.4	11.5	41.6
YH2	71.6	212.8	32.2	80.4	14.3	34.5
YH3	56.9	187.8	32.2	80.4	33.2	32.9
YH4	58.5	202.1	32.2	80.4	27.1	24.5
YH5	67.5	206.9	32.2	80.4	18.8	32.1
YH6	67.5	209.1	32.2	80.4	18.2	31.2
YH7	68.1	209.2	32.2	80.4	17.7	31.9
YH8	65.7	210.6	32.2	80.4	19.0	28.4
YH9	62.4	204.5	32.2	80.4	23.4	27.6
YH10	57.3	198.5	32.2	80.4	29.3	25.6
YH11	58.2	208.1	32.2	80.4	25.1	20.1
YH12	59.3	206.2	32.2	80.4	25.0	22.8
YH13	59.2	198	32.2	80.4	28.0	28.0
YH14	62.4	211.9	32.2	80.4	20.8	23.5
YH15	61.3	204.4	32.2	80.4	24.2	26.3
YH16	64.5	210.1	32.2	80.4	20.0	27.1

YH17	61.3	199.5	32.2	80.4	25.8	29.3
YH18	64.2	215.2	32.2	80.4	18.5	24.3
YH19	64.7	201.7	32.2	80.4	22.5	31.6
YH20	52.4	195.8	32.2	80.4	34.2	22.3
YH21	62.4	208.5	32.2	80.4	22.0	25.3
YH22	67.5	206.9	32.2	80.4	18.8	32.1
YH23	51.8	198.9	32.2	80.4	33.5	18.9
YH24	61.8	193.7	32.2	80.4	27.2	33.2
YH25	51	179.1	32.2	80.4	41.3	35.3
YH26	63.2	214.4	32.2	80.4	19.4	23.3
YH27	65.9	201.4	32.2	80.4	21.6	33.0
YH28	56.3	201	32.2	80.4	29.2	22.7
YH29	48.9	187.2	32.2	80.4	40.4	26.4
YH30	68.3	195	32.2	80.4	21.2	38.1
YH31	60.8	189	32.2	80.4	29.4	35.3
YH32	56.6	185.5	32.2	80.4	34.2	34.4
YH33	59	179.1	32.2	80.4	33.6	40.3
YH34	64.5	201.5	32.2	80.4	22.7	31.5
YH35	64.7	186.2	32.2	80.4	26.4	39.7
YH36	59	171.3	32.2	80.4	35.2	45.2
YH37	54.7	191.6	32.2	80.4	33.9	28.1
YH38	54.8	183.8	32.2	80.4	36.3	34.3
YH39	66.2	165.5	32.2	80.4	28.0	50.4
YH42	61.8	177.8	32.2	80.4	31.1	42.6
YH43	58	179.8	32.2	80.4	34.4	39.3
YH44	58.7	176.6	32.2	80.4	34.4	41.8
YH45	59.1	179.9	32.2	80.4	33.3	39.9
YH46	60.4	178.7	32.2	80.4	32.3	41.4
YH47	60.8	186.9	32.2	80.4	29.9	36.6
YH48	56	173.6	32.2	80.4	37.9	42.4
YH49	56.2	181.2	32.2	80.4	35.8	37.2
YH50	63.6	180	32.2	80.4	28.8	42.3
YH51	62.6	188.3	32.2	80.4	27.9	37.1
YH52	54.6	173.7	32.2	80.4	39.3	41.6
YH53	52.6	179.2	32.2	80.4	39.7	36.3
The Early Cretaceous (~110–131 Ma) Zenong Gp volcanic results from Cuoqin area reported by Chen et al.[6]						
DX1	50.4	350.1	32.2	80.4	315.7	41.8
DX2	51.7	340.5	32.2	80.4	319.7	34.8
DX3	46.9	354.3	32.2	80.4	311.0	43.5
DX4	52.5	2	32.2	80.4	315.5	51.1
DX5	69.6	326.4	32.2	80.4	339.9	39.4
DX6	65.5	333.4	32.2	80.4	334.6	39.6
DX7	56.7	348.8	32.2	80.4	322.5	43.8

DX8	70.7	322.8	32.2	80.4	341.6	38.8
DX9	56.9	345.7	32.2	80.4	323.4	41.8
DX10	50.9	342	32.2	80.4	318.5	35.5
DX11	40	350	32.2	80.4	305.5	35.7
DX13	62.7	352.1	32.2	80.4	328.4	48.0
C15	56.5	332.1	32.2	80.4	326.7	32.0
C16	61.9	331.5	32.2	80.4	331.7	36.0
C17	60.9	328.3	32.2	80.4	331.7	33.2
C18	69	300.4	32.2	80.4	346.2	28.8
C19	56.9	348.9	32.2	80.4	322.7	44.0
C20	56.7	342.9	32.2	80.4	323.9	39.8
The Early Cretaceous (~117–121 Ma) Dianzhong Fm volcanic results from Cuoqin area reported by Yang et al.[9]						
DZ14	66.1	234.1	32.2	80.4	10.5	20.1
DZ15	66.1	239.3	32.2	80.4	8.5	18.8
DZ17	79.1	204.6	32.2	80.4	10.0	43.9
DZ18	71.7	264.4	32.2	80.4	358.7	26.4
DZ19	61.9	308.7	32.2	80.4	338.9	22.7
DZ20	62.7	303.4	32.2	80.4	341.4	21.1
DZ21	62.6	307.2	32.2	80.4	339.9	22.9
DZ22	65.4	315.8	32.2	80.4	339.1	30.7
DZ23	65.2	307.7	32.2	80.4	341.5	26.6
DZ24	70.3	315.9	32.2	80.4	342.8	35.9
DZ25	70.8	307.9	32.2	80.4	345.2	33.6
DZ26	63.7	319.3	32.2	80.4	336.7	30.6
The Late Cretaceous (~92.5 Ma) volcanic results from Shiquanhe area reported by Yi et al.[23]						
xa161	60.8	193.9	32.2	80.4	28.0	32.2
xa162	66.0	203.5	32.2	80.4	20.9	32.1
xa163	73.8	224.4	32.2	80.4	10.0	34.1
xa164	76.0	259.2	32.2	80.4	0.3	33.3
xa166	75.8	303.3	32.2	80.4	349.7	38.1
xa172	50.0	189.7	32.2	80.4	38.5	25.1
xa176	51.7	198.2	32.2	80.4	33.8	19.4
xa181	55.7	214.5	32.2	80.4	24.0	12.3
xa182	56.5	202.4	32.2	80.4	28.5	21.9
xa183	52.2	209.1	32.2	80.4	28.7	11.0
The Late Cretaceous (~80 Ma) Chalicuo Group volcanic results from Yare area reported by Yi et al.[23]						
xy184	72.3	294.8	32.2	80.4	349.6	31.7
xy185	70.9	290.5	32.2	80.4	350.2	28.7
xy186	69.4	294.6	32.2	80.4	348.2	27.6
xy187	66.2	307.3	32.2	80.4	342.3	27.7
xy188	63.3	312.4	32.2	80.4	338.6	26.5
xy189	69.2	294.9	32.2	80.4	348.0	27.4
xy190	70.9	297.1	32.2	80.4	348.2	30.4

xy191	59.7	309.4	32.2	80.4	337.2	20.1
xy216	73.1	299.0	32.2	80.4	349.0	33.8
xy217	68.2	296.0	32.2	80.4	347.1	26.2
xy218	66.0	290.2	32.2	80.4	348.1	21
xy219	69.3	288.5	32.2	80.4	350.1	25.8
xy220	78.8	287.0	32.2	80.4	354.6	39.0
xy221	60.8	297.3	32.2	80.4	342.8	15.2
xy222	65.3	307.0	32.2	80.4	341.8	26.4
The Late Cretaceous (~68 Ma) Dianzhong Fm volcanic results from Shiquanhe area reported by Ma, Y.[10]						
LD1	46.9	200.2	32.2	80.4	36.6	11.6
LD2	57.3	178.5	32.2	80.4	35.4	39.8
LD3	42.4	187.9	32.2	80.4	45.6	19.2
LD4	40.0	188.3	32.2	80.4	47.4	16.1
LD5	44.8	176.7	32.2	80.4	47.9	33.1
LD6	43.8	172.1	32.2	80.4	50.4	36.8
LD7	36.3	168.4	32.2	80.4	58.9	35.8
LD8	40.4	177.8	32.2	80.4	51.5	28.5
LD9	40.1	176.7	32.2	80.4	52.2	29.5
LD10	43.9	186.4	32.2	80.4	45.0	22.4
LD11	45.4	186.5	32.2	80.4	43.7	23.7
LD12	46.5	188.2	32.2	80.4	42.1	23.1
LD13	44.5	173.7	32.2	80.4	49.2	35.8
LD14	53.5	182.7	32.2	80.4	37.9	34.1
LD15+16	51.2	182.9	32.2	80.4	39.9	32.2
LD17	47.5	191.1	32.2	80.4	40.1	21.2
LD18+19	47.8	183.7	32.2	80.4	42.7	28.7
LD20	49.8	178.1	32.2	80.4	42.7	35.3
LD21	52.3	174.2	32.2	80.4	41.4	40.0
LD22+23	51.4	179.4	32.2	80.4	40.8	35.3
LD24	48.3	183.8	32.2	80.4	42.2	29.0
LD25	49.9	176.1	32.2	80.4	43.2	37.1
LD26	45.1	175.9	32.2	80.4	47.9	34.1
LD27	43.9	179.1	32.2	80.4	47.8	30.0
LD28	48.1	179.6	32.2	80.4	43.8	32.8
LD30+31	47.7	177.4	32.2	80.4	44.9	34.5
LD32+33	49.3	177.4	32.2	80.4	43.4	35.6
LD34+35	49.5	171.6	32.2	80.4	44.9	40.6
LD36	52.8	174.9	32.2	80.4	40.8	39.8
LD37+38	51.6	173.7	32.2	80.4	42.3	40.0
LD39+40	47.7	174.7	32.2	80.4	45.8	36.9
LD41+42	51.6	173.6	32.2	80.4	42.3	40.1
LD46	61.0	225.7	32.2	80.4	16.2	14.6
LD47	33.4	170.4	32.2	80.4	60.8	31.5
LD48	70.7	208.1	32.2	80.4	16.1	35.1

LD49	24.0	169.6	32.2	80.4	69.7	25.0
The Late Cretaceous Jingzhushan Fm red beds results from Cuoqin area reported by Yang et al.[9]						
CQ1	36.6	340.8	32.2	80.4	306	22.7
CQ2	38.7	342.5	32.2	80.4	307.2	26.6
CQ3	42.7	341.4	32.2	80.4	311.2	28.7
CQ4	41.9	346.1	32.2	80.4	308.7	33
CQ5	39.6	346.2	32.2	80.4	306.5	31.4
CQ6	32.3	349.6	32.2	80.4	298.5	29.7
CQ7	37.7	343	32.2	80.4	306.1	26.3
CQ8	39.7	340.2	32.2	80.4	309	24.8
CQ9	44.8	343.3	32.2	80.4	312.4	32.3
CQ10	38	348.7	32.2	80.4	304.1	32.9
CQ11	42.7	345.3	32.2	80.4	309.7	32.7
CQ12	39.1	345.3	32.2	80.4	306.4	30
CQ13	26.6	352.4	32.2	80.4	292.1	28.8
CQ14	31.4	349.6	32.2	80.4	297.7	29
CQ15	32.9	348.2	32.2	80.4	299.6	28.5
CQ16	41.3	348.7	32.2	80.4	307.2	35.2
CQ17	38.3	346.3	32.2	80.4	305.3	30.5
CQ18	31.6	345	32.2	80.4	299.8	23.4
CQ19	33.7	349.9	32.2	80.4	299.6	31.2
CQ20	28.8	352.6	32.2	80.4	294	30.8
CQ21	64.2	318.5	32.2	80.4	337.3	30.8
CQ22	51.2	330.2	32.2	80.4	322.8	25.5
CQ23	67.9	327.7	32.2	80.4	338.1	38.6
CQ24	71	349.2	32.2	80.4	338	49
CQ25	61.4	355.4	32.2	80.4	326.5	49.3
CQ26	65	1.5	32.2	80.4	330.2	52.9
CQ27	64.7	347.3	32.2	80.4	331.2	46.2
CQ28	62.3	344.4	32.2	80.4	329.2	43.7
CQ29	70.9	346.6	32.2	80.4	338.1	48
CQ30	70.3	343.7	32.2	80.4	337.7	46.7
CQ31	80.5	319.4	32.2	80.4	350.9	45.5
CQ32	72.8	303.3	32.2	80.4	347.7	34.6
CQ33	66.8	320.6	32.2	80.4	338.8	34.4
The Late Cretaceous (~93–99 Ma) Dianzhong Fm volcanic results from Cuoqin area reported by Tang et al.[21]						
196	57.7	218.6	32.2	80.4	21	12.9
197	59.1	240.6	32.2	80.4	10	5.5
198	53.9	187.3	32.2	80.4	36	30.8
199	60.5	209.4	32.2	80.4	23	22.4
200	37.9	334.3	32.2	80.4	310	16
201	56.5	324.2	32.2	80.4	329.3	26.1
202	41.9	325.7	32.2	80.4	317.2	10.5

203	47.8	308.4	32.2	80.4	330.1	1.7
204	64.9	231.2	32.2	80.4	12.1	19
205	67	221.3	32.2	80.4	14.7	25.7
206	69.7	239.1	32.2	80.4	7.4	24.9
207	72.3	231.2	32.2	80.4	8.9	30.6
208	69.8	227.8	32.2	80.4	11.1	27.7
209	67.2	235.2	32.2	80.4	9.7	21.6
**The inclination-only mean of 100 Early Cretaceous sites from the western Lhasa terrane**						**34.2±1.8**
**The inclination-only mean of 108 Late Cretaceous sites from the western Lhasa terrane**						**30.0±2.2**
**The inclination-only mean of 208 Cretaceous sites from the western Lhasa terrane**						**32.1±1.5**
**The central Lhasa terrane**						
The Early Cretaceous (~120.2 Ma) Duoni Fm volcanic and sedmentary results from Naqu area reported by Li et al.[24]						
TL01	61.5	287.3	32.2	91.1	352.3	9.2
TL04	64.6	295.8	32.2	91.1	349.6	17.1
TL05	63.8	243.9	32.2	91.1	11.8	16.4
TL06	71.3	309.0	32.2	91.1	348.1	31.3
TL07	54.2	333.9	32.2	91.1	327.9	22.8
TL08	63.8	295.4	32.2	91.1	349.4	15.5
TL10	59.4	311.9	32.2	91.1	340.4	15.0
TL11	51.3	324.3	32.2	91.1	329.8	11.2
TL12	60.7	241.2	32.2	91.1	14.2	12.0
TL15	57.2	232.6	32.2	91.1	19.8	10.2
TL18	64.5	229.0	32.2	91.1	17.2	23.3
TL22	62.9	258.7	32.2	91.1	5.6	11.1
TL24	64.6	281.2	32.2	91.1	355.7	14.0
TL25	69.0	268.9	32.2	91.1	0.8	21.6
TND01	65.0	300.7	32.2	91.1	347.8	19.2
TND02	73.0	267.6	32.2	91.1	1.1	28.6
TND03	68.6	276.2	32.2	91.1	358.1	21
TND04	67.1	272.2	32.2	91.1	359.6	18.1
TND05	66.1	285.0	32.2	91.1	354.3	17.4
The Late Cretaceous (68-75 Ma) volcanic and red beds results from Linzhou area reported by Cao et al.[25]						
LZ2	66.4	301.9	32.2	91.1	347.9	21.9
LZ4	71.0	337.2	32.2	91.1	341.1	40.5
LZ5	72.9	313.2	32.2	91.1	348.0	34.5
LZ6	58.1	250.5	32.2	91.1	10.7	3.9
LZ7	58.8	243.9	32.2	91.1	13.7	7.5
LZ9	77.4	242.7	32.2	91.1	6.4	37.5
LZ10	64.8	270.2	32.2	91.1	0.4	13.8
LZ11	69.7	249.4	32.2	91.1	7.6	25.0
LZ12	78.4	208.4	32.2	91.1	11.5	44.7

LZ13	82.2	281.4	32.2	91.1	358.5	42.4
LZ19	61.2	301.2	32.2	91.1	345.9	13.0
LZ1	68.9	267.8	32.2	91.1	1.2	21.5
LZ3	73.5	268.0	32.2	91.1	0.9	29.4
LZ8	69.7	297.6	32.2	91.1	350.8	26
LZ14	66.9	254.8	32.2	91.1	6.4	19.2
LZ15	69.5	254.9	32.2	91.1	5.7	23.7
LZ16	71.7	282.4	32.2	91.1	356.4	26.9
LZ17	57.0	305.2	32.2	91.1	342.2	7.5
LZ18	60.4	255.2	32.2	91.1	7.8	7
LZ20	67.2	246.2	32.2	91.1	9.6	21.6
LZ21	69.0	216.6	32.2	91.1	17.9	34.2
The Late Cretaceous (K_2) red beds results from Dingqing area reported by Tong et al.[12]						
DQ1	70.1	281.0	32.2	91.1	356.6	24
DQ2	76.8	259.1	32.2	91.1	2.9	34.9
DQ3	67.5	281.5	32.2	91.1	356	19.4
DQ4	71.7	292.8	32.2	91.1	353.1	28.2
DQ5	69.3	300.6	32.2	91.1	349.7	26.1
DQ6	65.8	325.4	32.2	91.1	339.7	30.6
DQ7	77.6	262.5	32.2	91.1	2	35.9
DQ8	66.8	250.6	32.2	91.1	8.1	19.8
DQ9	70.3	259.7	32.2	91.1	3.9	24.5
DQ10	64.0	238.9	32.2	91.1	13.7	18.5
DQ11	66.0	231.7	32.2	91.1	15.3	24.4
DQ12	75.2	248.1	32.2	91.1	6	33.7
DQ13	59.4	305.6	32.2	91.1	343.1	11.8
DQ17	69.6	292.3	32.2	91.1	352.6	24.7
DQ18	70.0	244.4	32.2	91.1	9.1	26.5
The Late Cretaceous (K_2) volcanic and red beds results from Maxiang area reported by Sun et al.[19]						
XS1	75.7	291.7	32.2	91.1	354.7	34.1
XS2	74.4	352.9	32.2	91.1	342.3	47.6
XS3	62.7	332	32.2	91.1	335.3	30.7
XS4	59.6	325.4	32.2	91.1	335.1	23.2
XS5	68.3	313.1	32.2	91.1	345.1	28.6
XS6	73.2	358.5	32.2	91.1	340.5	49
XS7	73.4	326.9	32.2	91.1	345.2	39
XS8	81.9	253.3	32.2	91.1	2.7	42.3
XS9	78.9	329.8	32.2	91.1	349.5	44.3
XS10	63.1	319.6	32.2	91.1	339.7	24.4
XS11	65.8	339.3	32.2	91.1	336	37.4
XS12	73.7	292.6	32.2	91.1	353.8	31.3
XS13	69.7	311.5	32.2	91.1	346.5	29.9
XS14	71.9	6	32.2	91.1	338.6	51.3
XS15	61.5	4.4	32.2	91.1	326.8	48.5

XS16	78.2	296	32.2	91.1	354.7	38.1
XS17	66.9	330.5	32.2	91.1	339.1	34.1
XS18	48.7	205.8	32.2	91.1	37.5	18.7
XS19	58.1	245.9	32.2	91.1	13	5.5
XS20	59	215	32.2	91.1	26	23.6
The Late Cretaceous (K_2) volcanic results from Linzhou area reported by Tan et al.[20]						
32	72.3	180.6	32.2	91.1	20.7	49.9
31	70.5	176.6	32.2	91.1	23	50.9
30	72.1	163.1	32.2	91.1	21.2	55.4
29	75.6	244.9	32.2	91.1	6.7	34.7
28	74.3	248.2	32.2	91.1	6.3	32.4
27	64.5	191.9	32.2	91.1	27.7	42.2
26	66.5	179.7	32.2	91.1	27.3	48.9
22_25	72.3	172.5	32.2	91.1	21	52.5
18_21	61.3	173.8	32.2	91.1	33.9	50.5
13_17	59.2	160.5	32.2	91.1	37.2	57
12	65.7	207.2	32.2	91.1	23.1	35.2
11	69.7	213.7	32.2	91.1	18.1	36
10	68.3	189.4	32.2	91.1	24.2	45.2
9	69.6	230.3	32.2	91.1	13.7	29.9
8	54.7	187	32.2	91.1	38.5	39.8
7	68.8	185.3	32.2	91.1	24.2	47.1
6	75	189.7	32.2	91.1	17	47.7
5	45	181.2	32.2	91.1	49.7	39
4	76.3	215.7	32.2	91.1	12.3	41.4
3	65.6	190.7	32.2	91.1	26.8	43.4
2	71.6	192.3	32.2	91.1	20.3	45.5
The Late Cretaceous (K_2) red beds results from Linzhou area reported by Tan et al.[20]						
Sx1	68.3	294.1	32.2	91.1	351.5	22.9
Sx2	72.2	300.2	32.2	91.1	351.1	30.4
Sx3	72.5	284.6	32.2	91.1	355.8	28.4
Sx4	72.3	277.9	32.2	91.1	357.9	27.5
Sx5	62.6	326.9	32.2	91.1	336.8	27.7
Sx6	64.9	327.4	32.2	91.1	338.4	30.6
Sx7	66.6	315.3	32.2	91.1	343.4	27.1
Sx8	68.1	307.1	32.2	91.1	347	26.2
Sx9	66.8	301.5	32.2	91.1	348.3	22.4
Sx10	65.4	316.1	32.2	91.1	342.4	25.8
Sx11	63.1	306.9	32.2	91.1	344.4	18.5
Sx14	71.7	303.2	32.2	91.1	350	30.3
Sx15	68.2	335	32.2	91.1	339.1	37.4
Sx16	72.5	273.5	32.2	91.1	359.3	27.7
Sx17	73.3	278.3	32.2	91.1	357.9	29.2
Sx18	74.8	280.1	32.2	91.1	357.5	31.7

Sx19	72.6	310.4	32.2	91.1	348.5	33.4
Sx20	66.1	330	32.2	91.1	338.6	33.1
Sx21	72.8	333.5	32.2	91.1	343.4	40.5
Sx22	69	305.8	32.2	91.1	347.8	27.1
Sx23	68.7	287.1	32.2	91.1	354.1	22.3
Sx24	67.9	303.8	32.2	91.1	348	24.9
Sx25	59.5	320.1	32.2	91.1	337.1	19.8
Sx26	59.9	318.6	32.2	91.1	337.9	19.5
Sx27	63.8	319.7	32.2	91.1	340.1	25.4
Sx28	65	322.7	32.2	91.1	339.9	28.4
Sx29	65.8	304	32.2	91.1	346.9	21.7
Sx30	74.8	280.8	32.2	91.1	357.3	31.7
Sx31	71.7	275.9	32.2	91.1	358.4	26.4
Sx32	70.2	286.4	32.2	91.1	354.7	24.8
Sx33	72.9	304.4	32.2	91.1	350.3	32.3
Sx34	69.3	307.2	32.2	91.1	347.6	28
Sx37	69.9	296.8	32.2	91.1	351.2	26.1
SxB1	69.5	254.1	32.2	91.1	6	23.8
SxB2	72.3	266.4	32.2	91.1	1.5	27.4
SxB3	69.6	298.5	32.2	91.1	350.5	26.1
SxB4	65.6	312.5	32.2	91.1	343.7	24.6
SxB5	68.7	290.8	32.2	91.1	352.8	22.9
SxB6	71.9	282.6	32.2	91.1	356.3	27.2
SxB7	69.3	298.5	32.2	91.1	350.4	25.6
SxB8	63.5	209.2	32.2	91.1	24.4	32.1
SxB9	69.9	283.9	32.2	91.1	355.5	24
SxB10	69	299	32.2	91.1	350.1	25.2
The Late Cretaceous (K_2) red beds results from Linzhou area reported by Pozzi et al.[13]						
1	56.3	353.3	32.2	91.1	323.5	39.4
2	65.6	355.7	32.2	91.1	332.5	45.4
4	61	336	32.2	91.1	332.7	31.4
5	62.1	5.7	32.2	91.1	327.3	49.3
7	72.4	261.5	32.2	91.1	3	27.9
8	65.2	330.6	32.2	91.1	337.7	32.5
12	72.2	347.2	32.2	91.1	340.6	44.8
The Late Cretaceous (K_2) red beds results from Linzhou area reported by Westphal et al.[14]						
1	56.3	353.8	32.2	91.1	323.4	39.7
2	65.6	356.2	32.2	91.1	332.5	45.7
4	61.0	336.0	32.2	91.1	332.7	31.4
5	62.1	5.7	32.2	91.1	327.3	49.3
8	65.2	330.6	32.2	91.1	337.7	32.5
12	72.2	347.2	32.2	91.1	340.6	44.8
The Late Cretaceous (K_2) red beds results from Barda area reported by Achache et al.[15]						
13	65.8	324.7	32.2	91.1	339.9	30.3

14	68.2	308.1	32.2	91.1	346.7	26.7
17	52	322.1	32.2	91.1	331.3	10.5
18	63.1	328.2	32.2	91.1	336.8	29
19	58.7	326.5	32.2	91.1	334.1	22.8
21	72.2	329.9	32.2	91.1	343.6	38.8
The Late Cretaceous (K_2) red beds results from Barda area reported by Achache et al.[15]						
23	67.9	272.9	32.2	91.1	359.3	19.6
29	58.4	315.3	32.2	91.1	338.4	15.4
30	69.3	230.8	32.2	91.1	13.7	29.3
32	74.3	267.4	32.2	91.1	1	30.7
33	65.4	321	32.2	91.1	340.8	28.1
34	73.9	298.3	32.2	91.1	352.4	32.5
35	71.7	290.3	32.2	91.1	353.9	27.8
38	70.9	284	32.2	91.1	355.7	25.7
**The inclination-only mean of 166 Cretaceous sites from the central Lhasa terrane**						**30.0±2.2**
**The western Qiangtang terrane**						
The Early Cretaceous (Albian-Aptian) Longmucuo re sandstone results reported by Chen et al.[17]						
03	65.7	212.8	32.2	80.4	18.3	27.4
04	59.8	234.4	32.2	80.4	12.8	8.9
08	59.5	228.9	32.2	80.4	15.4	10.6
09	70.9	253.6	32.2	80.4	2.3	25.2
The Early Cretaceous (Albian-Aptian) Aksaichin red sandstone results reported by Chen et al.[17]						
41	66.5	273.6	32.2	80.4	354.7	18
42	64.6	264.5	32.2	80.4	358.2	13.5
44	65.4	267.3	32.2	80.4	357.1	15.2
45	52.6	232.5	32.2	80.4	16.5	-3.5
46	66.2	258.0	32.2	80.4	1	16.5
48	70.5	268.7	32.2	80.4	357.2	24.6
49	66.4	247.1	32.2	80.4	5.4	17.8
The Early Cretaceous (~104–111 Ma) Qushenla Fm volcanic results reported by Chen et al.[27]						
GZ23	78.3	339.6	32.2	80.4	346.8	48.3
GZ24-25	62.4	342.5	32.2	80.4	329.7	42.7
GZ26-28	77.8	329.6	32.2	80.4	347.2	45.8
GZ29	82.6	315.0	32.2	80.4	353.2	46.4
GZ30	75.4	301.3	32.2	80.4	349.8	37.2
GZ31	76.5	305.8	32.2	80.4	349.7	39.4
GZ32	77.5	258.2	32.2	80.4	0.5	35.6
GZ33-34	78.3	294.5	32.2	80.4	352.9	39.4
GZ35-36	77.6	43.8	32.2	80.4	350.1	60.8
GZ37	76.1	52.7	32.2	80.4	351.0	62.8
GZ38	79.5	62.3	32.2	80.4	355.6	61.0
GZ39-41	82.8	51.8	32.2	80.4	355.6	57.8
GZ42-43	69.7	346.7	32.2	80.4	336.7	47.6
GZ44-45	65.5	333.4	32.2	80.4	344.6	39.6

The Late Cretaceous–103.8 Ma Qushenla Fm Red beds (without E/I correction) results reported by Chen et al.[27]						
GZ1	45.9	350.2	32.2	80.4	311.2	39.5
GZ2	47.9	352.4	32.2	80.4	312.6	42.4
GZ3	43.6	336.6	32.2	80.4	313.9	24.4
GZ4	37.7	348.1	32.2	80.4	304.1	32.1
GZ5	37.7	350.0	32.2	80.4	303.3	34.2
GZ6	43.8	354.1	32.2	80.4	307.9	41.8
GZ7	40.5	351.9	32.2	80.4	305.3	37.9
GZ8	41.8	351.2	32.2	80.4	306.8	38
GZ9	48.1	351.0	32.2	80.4	313.2	41.3
GZ10	56.7	339.7	32.2	80.4	324.7	37.6
GZ11	40.5	352.0	32.2	80.4	305.3	38
GZ12	46.3	354.6	32.2	80.4	310.3	43.5
GZ13	51.3	348.8	32.2	80.4	317	41.2
GZ14	44.7	354.2	32.2	80.4	308.8	42.3
GZ15	55.2	330.8	32.2	80.4	326	29.8
GZ16	32.2	354.7	32.2	80.4	296.3	35.7
GZ17	39.4	345.5	32.2	80.4	306.6	30.5
GZ18	43.1	343.3	32.2	80.4	310.8	31
GZ19	41.1	348.3	32.2	80.4	307.2	34.7
GZ20	60.4	336.8	32.2	80.4	329	38.1
GZ21	44.3	349.8	32.2	80.4	309.7	38.2
GZ22	52.5	344.3	32.2	80.4	319.4	38.4
**The inclination-only mean of 47 Cretaceous sites from the western Qiangtang terrane**						**36.4±5.1**

Notes: Plat and Plon, latitude and longitude of pole; Rlat and Rlon, latitude and longitude of reference point; Dr and Ir, declination and inclination calculated for the reference point (32.2°N, 80.4°E) for the western Lhasa and Qiangtang terranes, and reference point (32.2ºN, 91.1ºE) for the central Lhasa terrane. The inclination-only mean is calculated following the method of Arason and Levi[50].

References

6. Chen, W. et al. Paleomagnetic results from the Early Cretaceous Zenong Group volcanic rocks, Cuoqin, Tibet, and their paleogeographic implications. *Gondwana Res.* **22,** 461–469 (2012).

9. Yang, T. et al. New insights into the India-Asia collision process from Cretaceous paleomagnetic and geochronologic results in the Lhasa terrane. *Gondwana Res.* **28,** 625–641 (2015a).

10. Ma Y. Paleomagnetism and Geochronology of Cretaceous Rocks from the Lhasa Terrane and the Tethyan Himalaya. *PhD thesis*, Beijing, China, China University of Geosciences. (in Chinese with English Abstract) (2016).

12. Tong, Y. et al. Paleomagnetism of the Upper Cretaceous red-beds from the eastern edge of the Lhasa Terrane: New constraints on the onset of the India-Eurasia collision and latitudinal crustal shortening in southern Eurasia. *Gondwana Res.* **48,** 86–100 (2017).

13. Pozzi, J. P., Westphal, M., Zhou, Y., Xing, L. & Chen, X. Position of the Lhasa block, South Tibet, during the late Cretaceous. *Nature* **297,** 319–321 (1982).

14. Westphal, M., Pozzi, J. P., Zhou, Y. X., Xing, L. S. & Chen, X. Y. Palaeomagnetic data about southern Tibet (Xizang) - I. The Cretaceous formations of the Lhasa block. *Geophys. J. R. astr. Soc.* **73,** 507–521 (1983).

15. Achache, J., Courtillot, V. & Zhou, Y. Palaeogeographic and tectonic evolution of southern Tibet since middle Cretaceous time: new paleomagnetic data and synthesis. *J. Geophys. Res.* **89,** 10311–10339 (1984).

17. Chen, Y., Cogné, J. P., Courtillot, V., Tapponnier, P. & Zhu, X. Cretaceous paleomagnetic results from western Tibet and tectonic implications. *J. Geophys. Res.* **98,** 17981–17999 (1993).

19. Sun, Z. et al. Palaeomagnetism of late Cretaceous sediments from southern Tibet:

Evidence for the consistent palaeolatitudes of the southern margin of Eurasia prior to the collision with India. *Gondwana Res.* **21,** 53–63 (2012).

20. Tan, X. et al. New paleomagnetic results from the Lhasa block: revised estimation of latitudinal shortening across Tibet and implications for dating the India-Asia collision. *Earth Planet. Sci. Lett.* **293,** 396–404 (2010).

21. Tang, X. et al. Paleomagnetism and Ar–Ar geochronology of Cretaceous volcanic rocks in the middle Lhasa terrane, China and tectonic implications. *Chin. J. Geophys.* **56,** 136–149 (2013) (in Chinese with English abstract).

22. Ma, Y. et al. Paleomagnetism and U-Pb zircon geochronology of Lower Cretaceous lava flows from the western Lhasa terrane: New constraints on the India-Asia collision process and intracontinental deformation within Asia. *J. Geophys. Res. Solid Earth* **119,** 7404–7424 (2014).

23. Yi, Z. et al. A quasi-linear structure of the southern margin of Eurasia prior to the India-Asia collision: First paleomagnetic constraints from Upper Cretaceous volcanic rocks near the western syntaxis of Tibet. *Tectonics* **34,** 1431–1451 (2015).

24. Li, Z., Ding, L., Song, P., Fu, J. & Yue, Y. Paleomagnetic constraints on the paleolatitude of the Lhasa block during the Early Cretaceous: Implications for the onset of India-Asia collision and latitudinal shortening estimates across Tibet and stable Asia. *Gondwana Res.* **41,** 352–372 (2017).

25. Cao, Y. et al. New Late Cretaceous paleomagnetic data from volcanic rocks and red beds from the Lhasa terrane and its implications for the paleolatitude of the

southern margin of Asia prior to the collision with India. *Gondwana Res.* **41,** 337–351 (2017).

27. Chen, W. et al. Combined paleomagnetic and geochronological study on Cretaceous strata of the Qiangtang terrane, central Tibet. *Gondwana Res.* **41,** 373–389 (2017).

33. Day, R., Fuller, M. & Schmidt, V. A. Hysteresis properties of titanomagnetites: Grain-size and compositional dependence. *Phys. Earth Planet. Inter.* **13,** 260–267 (1997).

34. Dunlop, D. J. Theory and application of the Day plot (Mrs/Ms versus Hcr/Hc): 2. Application to data for rocks, sediments, and soils. *J. Geophys. Res.* **107(B3),** 2057, doi:10.1029/2001JB000487 (2002).

50. Arason, P. & Levi, S. Maximum likelihood solution for inclination-only data in paleomagnetism. *Geophys. J. Int.* **182,** 753–771 (2010).
